# Three-Dimensional Genome Interactions Identify Potential Adipocyte Metabolism-Associated Gene *STON1* and Immune-Correlated Gene *FSHR* at the rs13405728 Locus in Polycystic Ovary Syndrome

**DOI:** 10.3389/fendo.2021.686054

**Published:** 2021-06-24

**Authors:** Can-hui Cao, Ye Wei, Rang Liu, Xin-ran Lin, Jia-qi Luo, Qiu-ju Zhang, Shou-ren Lin, Lan Geng, Si-kang Ye, Yu Shi, Xi Xia

**Affiliations:** ^1^ Center for Reproductive Medicine, Department of Obstetrics and Gynecology, Peking University Shenzhen Hospital, Shenzhen Peking University-The Hong Kong University of Science and Technology Medical Center, Shenzhen, China; ^2^ Department of Gynecologic Oncology, Tongji Hospital, Tongji Medical College, Huazhong University of Science and Technology, Wuhan, China; ^3^ Department of Critical Care Medicine, The Eighth Affiliated Hospital, Sun Yat-sen University, Shenzhen, China; ^4^ Department of Ultrasonography, Peking University Shenzhen Hospital, Shenzhen Peking University-The Hong Kong University of Science and Technology Medical Center, Shenzhen, China

**Keywords:** PCOS, three-dimensional genome analysis, rs13405728 locus, *STON1*, *FSHR*, *AR*

## Abstract

**Background:**

rs13405728 was identified as one of the most prevalent susceptibility loci for polycystic ovary syndrome (PCOS) in Han Chinese and Caucasian women. However, the target genes and potential mechanisms of the rs13405728 locus remain to be determined.

**Methods:**

Three-dimensional (3D) genome interactions from the ovary tissue were characterized *via* high-through chromosome conformation capture (Hi-C) and Capture Hi-C technologies to identify putative targets at the rs13405728 locus. Combined analyses of eQTL, RNA-Seq, DNase-Seq, ChIP-Seq, and sing-cell sequencing were performed to explore the molecular roles of these target genes in PCOS. PCOS-like mice were applied to verify the expression patterns.

**Results:**

Generally, *STON1* and *FSHR* were identified as potential targets of the rs13405728 locus in 3D genomic interactions with epigenomic regulatory peaks, with *STON1* (*P*=0.0423) and *FSHR* (*P*=0.0013) being highly expressed in PCOS patients. *STON1* co-expressed genes were associated with metabolic processes (*P*=0.0008) in adipocytes (*P*=0.0001), which was validated in the fat tissue (*P*<0.0001) and ovary (*P*=0.0035) from fat-diet mice. The immune system process (GO:0002376) was enriched in *FSHR* co-expressed genes (*P*=0.0002) and PCOS patients (*P*=0.0002), with *CD4* high expression in PCOS patients (*P*=0.0316) and PCOS-like models (*P*=0.0079). Meanwhile, *FSHR* expression was positively correlated with *CD4* expression in PCOS patients (P=0.0252) and PCOS-like models (*P*=0.0178). Furthermore, androgen receptor (*AR*) was identified as the common transcription factor for *STON1* and *FSHR* and positively correlated with the expression of *STON1* (*P*=0.039) and *FSHR* (*P*=4e-06) in ovary tissues and PCOS-like mice.

**Conclusion:**

Overall, we identified *STON1* and *FSHR* as potential targets for the rs13405728 locus and their roles in the processes of adipocyte metabolism and *CD4* immune expression in PCOS, which provides 3D genomic insight into the pathogenesis of PCOS.

## Introduction

Polycystic ovary syndrome (PCOS) is a gynecological endocrine disorder that has been one of the leading causes in female infertility (1, 2). It is characterized by hormonal imbalance and ovarian dysfunction, with symptoms of hyperandrogenism, anovulation, and polycystic ovarian morphology (3). PCOS occurs in 4-8% of women worldwide and affects 6-12% (approximately 5 million) of reproductive age women in the United States (4). Moreover, women with PCOS have been reported to be at higher risk for hypertension, insulin resistance (IR), diabetes, psychiatric disorders, dyslipidemia, and cancers (4, 5).

The high heritability of PCOS as a genetic trait has been reported to account for 70% of the incidence of the disorder (2). The application of genome-wide association studies (GWAS) in large case-control cohorts has successfully supported the discovery and characterization of PCOS susceptibility loci. Some loci are close to genes that play a role in reproductive processes or metabolic dysfunction, such as rs13405728, rs2268361, and rs2349415 to follicle stimulating hormone receptor (*FSHR*), rs11031006 to *FSHB*, rs2059807 to *INSR*, and rs2272046 to *HMGA2* (6). The growing list of PCOS risk loci contributes to the understanding of the etiological pathways and processes of the syndrome and reveals the relative homology genetic basis of PCOS (6, 7). However, over 95% of GWAS-associated risk loci were found to be localized in the non-coding regions (8), while long distances exist between risk loci and target genes (9), making their pathological roles in PCOS unclear. rs13405728 has been identified as the most susceptibility locus for PCOS on 2p16.3 in Han Chinese women (7, 10) and Caucasian women (11). However, the target genes of rs13405728 and the roles of such risk locus in the development of PCOS remain to be determined.

Comprehensive and direct long-range mapping of regulatory elements and target genes is crucial to systematically understand the transcriptional regulation of human diseases (12, 13). Since researchers have provided insight into the three-dimensional (3D) structural genome of disease by mapping the interactions between baits and target genes using high-throughput and long-range approaches, such as high-throughput chromosome conformation capture (Hi-C), or Capture Hi-C (14, 15), it is increasingly evident that alternative chromatin interactions are responsible for the gene dysregulation and biological phenotype in human disease or complex traits (13). For example, SNPs in intron 19 of *CLEC16A* are associated with the expression of *DEXI* (16), rs6927172 in region 6q23 in autoimmune diseases is associated with the increased expression of *IL20RA* (17), and rs9349379 in vascular diseases is associated with the expression of *EDN1* (18).

In this study, 3D structural genomic analysis from Hi-C and Capture Hi-C, expression profiling of PCOS patients and PCOS-like models, ChIP-Seq analysis of androgen receptor (*AR*) in *STON1* and *FSHR*, and single-cell sequencing of ovary tissue were used to synthesize the 3D interactions, adipocyte metabolism association with *STON1*, and *CD4* immune association with *FSHR* at the rs13405728 locus in PCOS.

## Material and Methods

### PCOS-Like Models and Mouse Tissue Acquisition

The animal study (C57BL/6) was performed with the approval of the Ethics Committee of the Peking University Shenzhen Hospital (PKUSH) and performed in Shenzhen Peking University-The Hong Kong University of Science (PKU-HKUST) and Technology Medical Center. The PCOS-like models (testosterone-treated and high-fat diet) of research were followed by the previous studies (19–21). Fat mice were treated with a fat diet (with 60% fat), while control mice were treated with a normal diet. Testosterone-treated mice used dihydrotestosterone release pellet (Dow Corning, Midland, MI, USA, 10 mg, S4757, Selleck) with a hypodermic way for 90 days. All performances were conducted under the Animal Welfare Act (AWA) and the Administrative Procedure Act (APA) Guidelines. Hematoxylin and eosin (H&E) staining of representative ovaries and quantitative analysis of cystic follicles were shown in **Supplementary Figures 1A, B**.

### Immunohistochemistry (IHC)

Mouse tissue was prepared as formaldehyde-fixed and paraffin-embedded (FFPE) after collection and rinse. 4 μm sections were obtained from FFPE tissue with a microtome and then de-paraffinization and antigen retrieval were completed. To prevent background staining and false-positive results, endogenous peroxidase was inactivated by 3% H_2_O_2_ and any non-specific binding proteins were quenched by bovine serum albumin (BSA, 5%, Servicebio). Primary antibody against FHSR (A3172, ABclonal, 1:100), *STON1* (PA5-75314, Invitrogen, 1:50) and AR (A19611, ABclonal, 1:100) was applied at 4°C overnight. After rinsing, the samples were treated with biotinylated secondary anti-rabbit immunoglobins and peroxidase-conjugated streptavidin, incubating at room temperature for 1 hour. The score of results was evaluated *via* Image-Pro Plus.

### Hi-C Maps and Virtual 4C Analysis

Hi-C experiments of ovary tissue were downloaded from GSM2322546 (22), which were performed by HindIII restriction enzyme using the Hi-C “dilution” protocol (9). NHEK Hi-C data were downloaded from GSE63525 (23), which were performed by MboI restriction enzyme using the *in situ* Hi-C Protocol. Comparative Hi-C map between ovary tissue and GM12878 (as control) was generated by 3DIV tool (http://kobic.kr/3div), an online interaction viewer for Hi-C interactions (24). Interaction genes of rs13405728 were shown in **Supplementary Table 1**. rs13405728 was used as the bait with a 500Kb interaction range on chromosome 2 (chr2: 48478159-49478159). Virtual 4C map was generated from the ovary Hi-C data with the viewpoint of chr2:47978158-49978158 *via* 3D Genome Browser (http://3dgenome.org) (25), rs13405728 was used as the bait with 500Kb interaction range. All data was processed by a custom pipeline with the hg19 reference genomes.

### Capture Hi-C and DHS Linkage Analysis

Capture Hi-C data of the ovary tissue were downloaded from GSM2322546 (22). Capture Hi-C analysis was performed by 3D Genome Browser (http://3dgenome.org) with the default settings (25). rs13405728 was used as the bait with a 500Kb interaction range on chromosome 2. DNase hypersensitive site (DHS)-linkage profiling was performed as described previously (26), which was performed by 3D Genome Browser (http://3dgenome.org) with the default settings (25). DHS-linkage computed the Pearson correlation coefficients for all distal DHSs with gene proximal DHS, which was based on the tissue-specificity (25).

### Compartment A/B Analysis

A/B compartment of cells (Normal cervical cells and cervical carcinoma) was downloaded from Genome Sequence Archive (GSA, http://bigd.big.ac.cn/gsa/), with the link number CRA001401. A Compartment matrix was performed as described previously (27). A/B compartment matrix was constructed using Integrative Genomics Viewer (v2.5.0), with region chr2: 47978158-49978158 (GRCh37/hg19).

### Chromatin Immunoprecipitation Sequencing (ChIP-Seq) Analysis

ChIP-Seq (H3K36me3, H3K4me1, H3K9me3, and H3K27ac) of the ovary was explored in Roadmap Epigenomics Project (http://www.roadmapepigenomics.org/data/), an online public resource of epigenomic maps for primary *ex vivo* tissues (release 9). Peak annotation of H3K36me3, H3K4me1, H3K9me3, and H3K27ac were integrated from adult human ovaries. Genome region were chr2:48478158-49478158 (GRCh37/hg19). ChIP-Seq data AR of primary tissues were downloaded from androgen receptor programming of human tissue (GSE56288 and GSE70079). The ChIP-Seq results were viewed using the UCSC Genome Browser.

### Data for Single-Cell Sequencing

Single-cell sequencing data of mouse ovary was downloaded from Mouse Cell Atlas (MCA) (28), which were performed by following the Microwell-seq protocol. The pooled data of mouse tissue included embryo, brain, heart, intestine, kidney, liver, lung, pancreas, stomach, testis, uterus, bladder, spleen, thymus, and prostate, and the cells were mapped into 99 clusters in tSNE plot. 4363 cells of adult mouse ovary were sequenced to analyze the expression of different cells. All these cells were clustered into 14 types with the tSNE dimension reduction method. The heatmap of cell types was conducted by Mouse Cell Atlas (MCA2.0, http://bis.zju.edu.cn/MCA/index.html). The expression of *Fshr* was explored in different cell types, with the mean expression of the cluster. The results were read by transcripts per kilobase of exon model per million mapped reads (TPM, **Supplementary Table 1**).

### Expression Data Acquisition and Correlation Analysis

Expression data (GSE156895, GSE145461, GSE114419, GSE138518, GSE8157, GSE124707, GSE135917, and GSE43322 profiling data, **Supplementary Table 2**) were downloaded from the Gene Expression Omnibus (GEO, http://www.ncbi.nlm.nih.gov/geo). RNA-seq data of ovary tissue was downloaded from the Roadmap Epigenomics Project (http://www.roadmapepigenomics.org/data/). In GSE135917 and GSE43322, *STON1* probe expressions were extracted and analyzed for Pearson’s correlation with BMI of samples in different groups. In GSE8157 and GSE124707, *STON1* expression signals were extracted and performed for Pearson’s correlation with *CD4* expression signals in different groups. Correlation analyses of *STON1* and *AR*, *FSHR* and *AR* were performed in Gene Expression Profiling Interactive Analysis (GEPIA, http://gepia.cancer-pku.cn/index.html) using the ovary tissue data from Genotype-Tissue Expression (GTEx) project. Data of PCOS patients were from Chinese infertility women (GSE145461, GSE114419), Han Chinese infertility women (GSE138518), and Caucasian infertility women (GSE8157). The race of PCOS patients used to explore phenotype was consistent with the population of rs13405728 locus. In addition, the main phenotypes of the four data were listed in **Supplementary Table 3**, including age, BMI (Kg/m2), LH(IU/L), FSH(IU/L), testosterone (ng/dL), and so on.

### Expression Quantitative Trait Loci (eQTL) Analysis

eQTL analysis was performed in QTLbase (http://mulinlab.org/qtlbase/index.html) and GTExPortal (https://gtexportal.org/home/). To investigate the effect of rs13405728 locus candidate on target gene expression, eQTL analysis was performed *via* GTEx project for single-tissue eQTL and QTLbase for Cis-eQTL, which were used to evaluate the expression changes and understand the biological function of genetic polymorphism.

### Co-Expression Networks

Co-expression networks of *STON1* and *FSHR* were performed in GeneMANIA (29) (http://genemania.org), an online tool including 2277 associated networks mapped to 163599 genes from 9 organisms. *STON1* (ENSG00000243244) and *FSHR* (ENSG00000170820) were used as input genes. Co-expression networks of *STON1* and *FSHR* were explored in humans (*Homo sapiens*) with the default settings. Co-expression networks included physical interactions, co-expression, predicted interactions, co-localization, pathway interactions, genetic interactions, and shared protein domains.

### Gene Set Enrichment Analysis and Enrichment Analysis in PaGenBase

Gene Ontology (GO) analysis was performed to annotate the gene function and biological characteristics in interaction networks using Gene Ontology consortium (http://www.geneontology.org/). GO analysis of co-expression genes and differentially expressed genes (DEGs) in PCOS was performed by Metascape (30) (https://metascape.org). Gene lists of co-expressed genes of *STON1* and *FSHR* were analyzed in Metascape (https://metascape.org) *via* the PaGenBase tool, which was a pattern gene database for understanding the gene function (31).

### Transcription Factor (TF) Analysis

An online pipeline for TF analysis, Toolkit for Cistrome Data Browser (32) (http://dbtoolkit.cistrome.org/), was used to construct the hierarchical TFs of *STON1* and *FSHR*. Genome used the human/hg38, the half-decay distance to the transcription start site was 10kb, and data type in Cistrome was transcription factor and chromatin regulator. *STON1* (chr2:48530168-48598514, NM_001198595) and *FSHR* (chr2:48953160-49154526, XM_011532734) were used as the input genes. The top 20 factors are shown in the plot. Regulatory potential (RP) represented the score to estimate how possible the TF could regulate the gene. Y-axis is the RP score, X-axis is different factors. Dots in the X-axis represent the same factors.

### Statistical Analysis

Data were presented as mean ± standard deviation (SD). All statistical analyses were performed on the statistical package of GraphPad Prism 6 (v6.02). Pearson’s correlation coefficient was used for the evaluation of the correlation between *FSHR* and *CD4*, *STON1* and BMI, *AR* and *STON1*, *AR* and *FSHR*. The Student’s t-test was used for the assessment of the difference among different groups. All the parameters would be considered statistically significant with a *P*-value<0.05.

## Results

### Hi-C Maps and Epigenomic Peaks in the Region of the rs13405728 Locus

**Figure 1:**
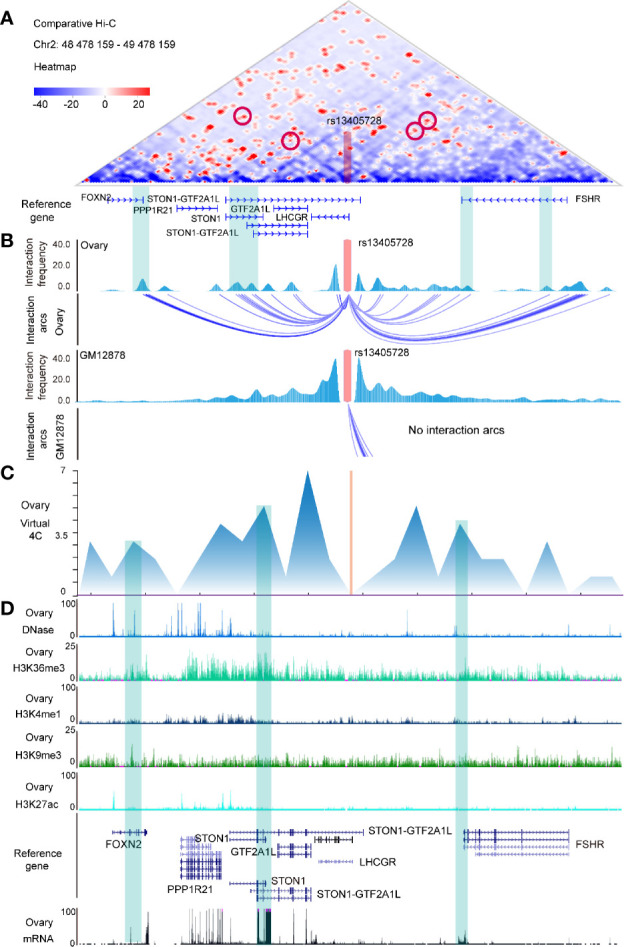
Hi-C maps and epigenomic peaks in the region of the rs13405728 locus. **(A)** Comparative Hi-C map between ovary tissue and GM12878 in the Chr2:48478159-49478159 region with the rs13405728 locus labeled. **(B)** Interaction frequency and interaction arcs of the rs13405728 locus from ovary tissue and GM12878. **(C)** Virtual 4C interactions of the ovary in the Chr2:48478159-49478159 region. **(D)** DNase-Seq, ChIP-Seq (H3K36me3, H3K4me1, H3K9me3, and H3K27ac), and RNA-Seq peaks of the ovary. Reference genes and the rs13405728 locus are shown. Hi-C high-through chromosome conformation capture.

We explored Hi-C interactions of ovary tissue (**Supplementary Table 3**) and performed a comparative Hi-C map between ovary tissue and GM12878 (as control) (**Figure 1A**), finding that *FOXN2*, *STON1-GTF2A1L*, *STON1*, *GTF2A1L*, and *FSHR* were the putative targets of the rs13405728 (Chr2:48978158) locus with interaction arcs in ovary tissue (**Figure 1B**). We then explored Hi-C maps and TADs of ovary tissue and NHEK cells (normal epithelium) in the region of rs13405728 (**Supplementary Figures 2A, B**) and found that rs13405728, *STON1*, *LHCGR*, *STON1-GTF2A1L*, and *FSHR* tended to be in the same TAD. In addition, such domain of rs13405728 was identified as B compartment in Hi-C data of different cells (**Supplementary Figure 2C**).

Virtual 4C signal of ovary tissue was used to analyze the interactions between rs13405728 and target genes (**Figure 1C**). DNase-Seq, ChIP-Seq of H3K36me3, H3K4me1, H3K9me3, and H3K27ac and mRNA data were characterized to identify the epigenomic modulation of ovary tissue. We found the epigenomic peaks and expression peaks in *FOXN2*, *STON1*, *STON1-GTF2A1L*, *GTF2A1L*, and *FSHR* (**Figure 1D**).

### Expression Analysis Between the rs13405728 Locus and Potential Target Genes

**Figure 2:**
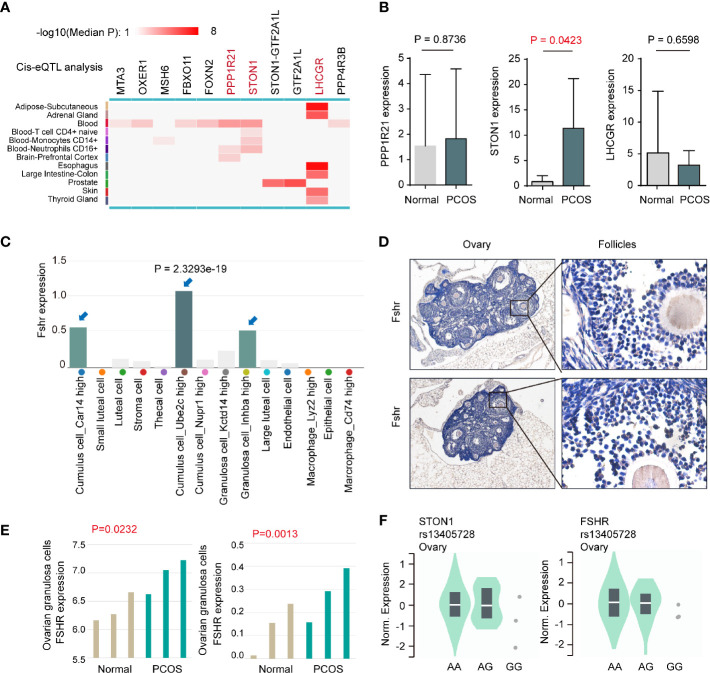
Expression analysis between the rs13405728 locus and potential target genes. **(A)** Cis-eQTL analysis of potential target genes around the rs13405728 locus region (+/- 10Mb region) from 12 tissues, the color bar is shown. **(B)** Gene expression in PCOS patients and normal patients from the GSE145461 dataset. **(C)** Fshr expression in ovarian cell clusters from ovary single-cell sequencing data. 14 clusters are shown. **(D)** IHC staining of Fshr in C57BL/6 ovary tissue. **(E)** Fshr expression of ovarian granulosa cells in PCOS patients and normal patients from GSE114419 and GSE138518 datasets. **(F)** Single-tissue eQTL analysis of *STON1* and *FSHR* at the rs13405728 locus from normal ovary tissue. PCOS, polycystic ovary syndrome; eQTL, expression quantitative trait loci.

To further explore the molecular patterns between rs13405728 and target genes, we conducted Cis-eQTL analysis around the rs13405728 locus region (+/- 10Mb region) from 12 tissues (**Figure 2A**). We further investigated whether these genes were changed in PCOS patients from GEO datasets, comparing with normal patients. *PPP1R21*, *STON1*, and *LHCGR* were found to be associated with SNP of rs13405728. In PCOS from the GSE145461 dataset, only *STON1* (*P* = 0.0423) was elevated in PCOS patients, with non-significance in *PPP1R21*, *LHCGR*, *FOXN2*, *STON1-GTF2A1L*, and *GTF2A1L* (**Figure 2B** and **Supplementary Figure 3A**). Although *LHCGR* was reputed as target gene for rs13405728 locus, there was no expression differences in PCOS patients. Additionally, *STON1* was identified as the target gene in Capture Hi-C interactions in ovary tissue, which was not found in blood control cells (CD4+ T cells and CD8+ T cells, B cells as control cells, **Supplementary Figure 3B**).

When comparing the expression of *FSHR* in human tissues (**Supplementary Figure 3C**), *FSHR* expression was specific to the ovary and testis. Single-cell sequencing data of ovary tissue was then mapped and found that *FSHR* highly expressed in Cumulus cell_Ube2c high cluster (*P* = 2.3293e-19), Granulosa cell_Inhba high cluster, and Cumulus cell_Car14 high cluster (**Figure 2C**). Such expression patterns were validated in mouse ovary tissues (**Figure 2D**). In ovarian granulosa cells of patients, the expression of *FSHR* was found to be higher in PCOS patients than normal patients, with GSE114419 (*P* = 0.0232) and GSE138518 (*P* = 0.0013) datasets (**Figure 2E**). In addition, we performed single-tissue eQTL analysis of *STON1*, *FSHR*, and rs13405728 SNP in ovary tissue (**Figure 2F**).

### *STON1* Was Associated With Metabolic Processes in Adipocytes and Highly Expressed in Mouse Fat and Ovary Tissue From Fat-Diet Mice

**Figure 3:**
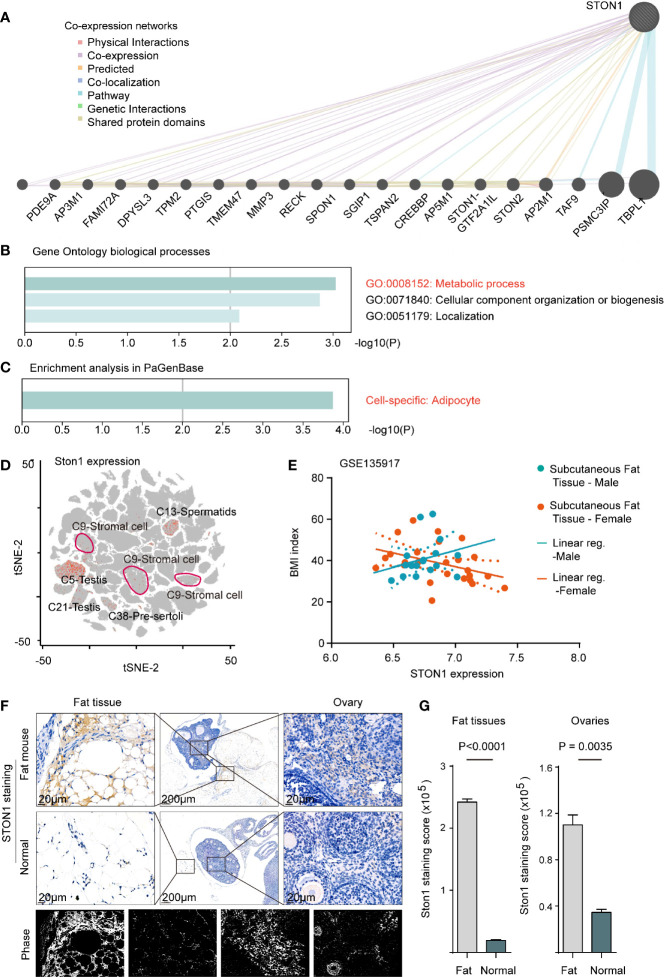
*STON1* was associated with metabolic processes in adipocytes and highly expressed in mouse fat and ovary tissue from fat-diet mice. **(A)** Co-expression networks of *STON1*, co-expressed genes and interactions are indicated. **(B)** GO enrichment analysis of *STON1* co-expressed genes. **(C)** Cell-specific analysis of *STON1* co-expressed genes from PaGenBase. **(D)** tSNE map of *STON1* in pooled mouse tissues from mouse single-cell sequencing data (Han et al., 2018), *STON1* expression is shown with cluster labeled. **(E)** Correlation analysis between *STON1* expression and BMI in males and females from GSE135917 dataset. IHC staining **(F)** and staining score **(G)** of *STON1* in fat tissue and ovary tissue from fat-diet mice. GO, Gene Ontology; tSNE, t-distributed stochastic neighbor embedding; BMI, body mass index; IHC, immunohistochemistry.

Since the biological roles of *STON1* in PCOS were unclear, we performed co-expression networks of *STON1* in the public dataset to analyze the molecular function of *STON1* in cells (**Figure 3A**). GO biological process analysis demonstrated that co-expressed genes of *STON1* were associated with metabolic processes (GO:0008152, P = 0.0008), cellular component organization or biogenesis (P = 0.001), and localization (P = 0.007) (**Figure 3B**). Enrichment analysis in PaGenBase showed that cell-specific enrichment of the networks was in adipocytes (P = 0.0001, **Figure 3C**).

To further explore the expression of *STON1* in the single-cell pattern, we performed the single-cell transcriptional analysis in the pooled data with 15 mouse tissues (see Methods). *STON1* was found in the reproductive gland (Cluster5, Cluster6, Cluster13, Cluster21, Cluster38) and stromal cells (Cluster9) (**Figure 3D** and **Supplementary Figures 4A, B**). In the GSE135917 dataset, the correlation tendency between *STON1* expression of subcutaneous fat and body mass index (BMI, kg/m^2^) was contrary between males and females (**Figure 3E**). In GSE155489, the expression of *STON1* was higher in PCOS than in normal control (**Supplementary Figure 4C**, P = 0.0391). Importantly, IHC staining of *STON1* was higher in fat diet mice, both in fat tissue (*P* < 0.0001) and ovary tissue (P = 0.0035), than normal diet mice (**Figures 3F, G**).

### *FSHR* Was Associated With The Immune System Processes And Positively Correlated With *CD4* Expression in PCOS Patients and PCOS-Like Models

**Figure 4:**
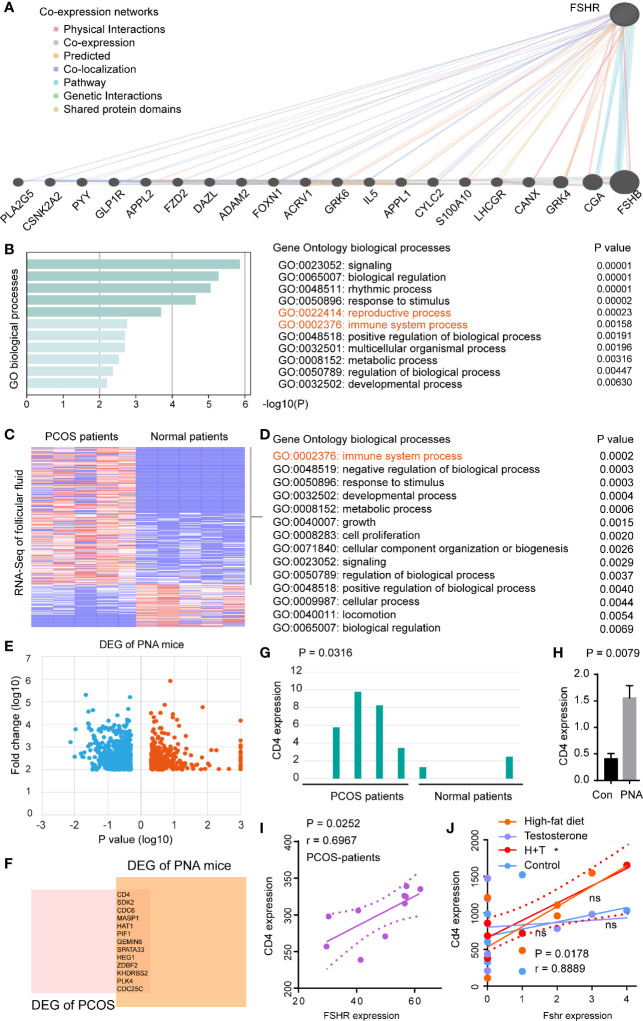
FSHR was associated with the immune system processes and positively correlated with CD4 expression in PCOS patients and PCOS-like models. **(A)** Co-expression networks of FSHR, co-expressed genes and interactions are indicated. **(B)** GO enrichment analysis of FSHR co-expressed genes. Heatmaps of DEGs **(C)** and GO enrichment analysis of upregulation DEGs **(D)** in the GSE145461 dataset. **(E)** Volcano plot of DEGs between the two groups of samples in GSE156895 dataset. Red spots indicate the up-regulated genes, blue spots indicate the down-regulated genes. **(F)** Venn diagram of upregulation DEGs between GSE145461 and GSE156895 datasets. Bar chart of CD4 expression between the two sets of samples in GSE145461 **(G)** and GSE156895 **(H)**. Correlation analysis of FSHR expression with CD4 expression in GSE8157 **(I)** and GSE124707 **(J)**. DEGs, differentially expressed genes; PNA mice, prenatally androgenized mice; H, high-fat diet; T, testosterone-treated. * < 0.05; ns, no significance.

To evaluate the molecular basis of *FSHR* in PCOS, we firstly performed co-expression networks of *FSHR* (**Figure 4A**). GO enrichment analysis demonstrated that co-expressed genes of *FSHR* were associated with the reproductive process (GO:0022414, *P* = 0.0002) and the immune system process (GO:0002376, *P* = 0.0002) (**Figure 4B**). The immune system process (GO:0002376, *P* = 0.0002) was found to be enriched in the differential expressed genes (DEGs) of follicular fluid between PCOS and normal patients (**Figures 4C, D**).

The hyperandrogenic phenotype was reported to be an important molecular mechanism of PCOS (33), thus prenatally androgenized (PNA) mice were conducted to analyze the DEGs between PCOS-like mice and normal control (**Figure 4E**). High expression of CD4 et al. was found in both PCOS patients (*P* = 0.0316) and PCOS-like mice (*P* = 0.0079) (**Figures 4F–H**). Furthermore, *FSHR* was found to be positively correlated (*P* = 0.0252, r = 0.6967) with *CD4* expression in PCOS patients (**Figure 4I**), and in PCOS-like (*Macaca mulatta*) model (*P* = 0.0178, r = 0.8889, **Figure 4J**).

### AR Was Identified as the Common Transcription Factor of *STON1* and *FSHR* and Positively Correlated With *STON1* and *FSHR* Expression in Ovary Tissues

**Figure 5:**
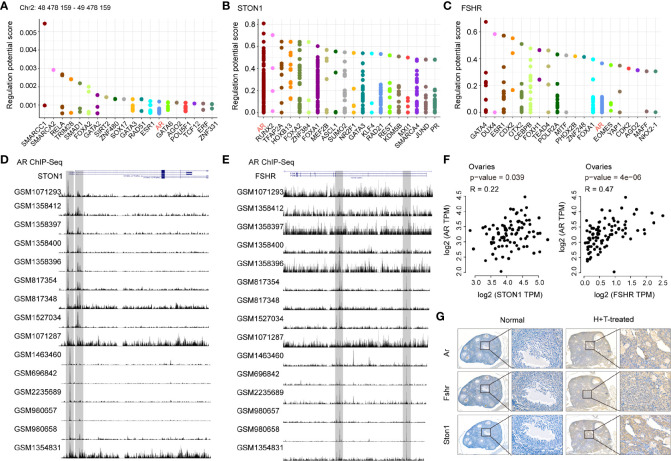
AR was identified as the common transcription factor of *STON1* and FSHR and positively correlated with *STON1* and FSHR expression in ovary tissues. The top 20 transcription factors for the region of the rs13405728 locus (Chr2: 48478159-49478159) **(A)** *STON1* **(B)** and FSHR **(C)**, regulatory potential (RP) represented the score to estimate how likely the TF could regulate the gene. Y-axis is the RP score, X-axis is different factors. Dots in the X-axis represent the same factors. ChIP-Seq peaks of AR in *STON1* **(D)** and FSHR **(E)** from androgen receptor programming of human tissues (GSE56288 and GSE70079). Correlation analysis between the expression of AR and *STON1*, AR and FSHR **(F)** in ovary tissues from GTEx dataset. **(G)** IHC staining of Ar, Fshr, and *STON1* in normal mice and high-fat diet and testosterone-treated mice. AR, androgen receptor; GTEx, Genotype-Tissue Expression; H+T, high-fat diet and testosterone-treated.

Given the increased expression of *STON1* and *FSHR* in PCOS patients and PCOS-like models, we hypothesized a potential role of *STON1* and *FSHR* in PCOS and explored the high expression mechanism underlying PCOS. We then performed transcription factors (TFs) analysis in the region of the rs13405728 locus (Chr2: 48478159-49478159) and gene regions of *STON1* and *FSHR* (**Figures 5A–C** and **Supplementary Tables 4**–**6**). AR was the only TF among them, *AR* and *FOXA1* were found to be the same TFs of *STON1* and *FSHR* (**Supplementary Figure 5A**), Further ChIP-Seq analysis of *STON1* and *FSHR* showed the modulation peaks of *AR* in primary tissues (**Figures 5D, E**). Moreover, the expression of *AR* was found to be positively correlated with the expression of *STON1* (*P* = 0.039, r = 0.22) and *FSHR* (*P* = 4e-06, r = 0.47) in normal ovary tissues (**Figure 5F**). However, the correlations were not found in *FOXA1* (**Supplementary Figure 5B**). Meanwhile, the expression of Ar, Fshr, and *STON1* were elevated in testosterone-treated and high-fat diet mice than normal mice (**Supplementary Figure 5C**), and their expression showed positive correlations (**Figure 5G**).

## Discussion

Since the rs13405728 locus has been identified as a common risk locus of PCOS (**Supplementary Figure 6**) in Han Chinese women (7, 10) and Caucasian women (11), it was necessary to identify target genes at the rs13405728 locus based on alternative genome conformation in the development of PCOS. We mapped Hi-C interactions, Capture Hi-C interactions, and virtual 4C interactions from the ovary tissue, and identified the potential targets at the rs13405728 locus. In addition, we explored the changes in expression of potential targets in PCOS patients and PCOS-like models, comparing with the normal patients and normal control, and identified *STON1* and *FSHR* as the most functional targets at the rs13405728 locus in PCOS. The Hi-C approach holds the advantages of capturing long-range interactions across the whole human genome (9), which is entirely useful for understanding the genetic trait with high heritability in the development of PCOS (2).

*STON1* has been reported to be involved in spermatogenesis of the mouse models (34), in accordance with our findings that *STON1* expressed in the reproductive gland and stromal cells in single-cell sequencing patterns, which was validated in adipocytes and ovaries of the high-fat diet mouse models. Our results found an opposite tendency of correlations between *STON1* and BMI in male and female adipocytes. In male adipocytes, BMI was positively correlated with *STON1* expression. In PCOS, high BMI is a common characteristic and was a predictor of hyperandrogenism (35), consisting of the findings that *STON1* was highly expressed in PCOS and PCOS-like models. These results suggested that high *STON1* expression may be responsible for the hyperandrogenic phenotype in PCOS patients with dysregulated metabolic phenotypes.

Currently, PCOS is also reputed as an autoimmune disorder with high autoantibodies recorded in long-term clinical management (36). In our findings, the immune system processes were enriched in PCOS patients, with a high *CD4* expression phenotype in PCOS patients and PCOS-like models. In addition, *FSHR*, a receptor for *FSH*, plays a role in the development of follicles, maturation of the oocyte, and regulation of steroidogenesis and may be an important candidate gene for PCOS (37). However, the role of *FSHR* in the development of PCOS is unclear. Here, we showed an enrichment of the immune system processes and reproductive processes in *FSHR* co-expressed genes, following a positive correlation between *CD4* and *FSHR* both in PCOS patients and PCOS-like models. These results are supported by the findings that PCOS had lower global DNA methylation in monocytes, T helper cells, T cytotoxic cells, and B cells (38).

The biochemical and clinical changes of hyperandrogenism (high levels of androgen) are important phenotypes of PCOS, which was associated with anovulation and menstrual dysfunction (3). Therefore. prenatally androgenized (PNA) models (39) or testosterone-treated models (20) were used as PCOS-like models for hyperandrogenism basis, which would highly increase the expression of *AR* (40). In our findings, AR was identified as the common TF of *STON1* and *FSHR* and positively correlated with their expression in ovary tissues. These results suggested the underlying interactions of hyperandrogenism, *AR*, *STON1*, and *FSHR* in the development of PCOS.

Insulin resistance is reputed as a key element contributing to the pathogenesis of PCOS patients (41). Recent studies have identified some candidate genes related to PCOS susceptibilities, such as the processes of insulin secretion and action in cells (42, 43). Therefore, we analyzed the expression levels of candidate genes in PCOS patients and PCOS-like models compared with normal patients and control. *IGF1* and *IGF1R* showed differential expression between PCOS and normal patients (**Supplementary Figure 7A**). *Igf1*, *Igfbp1*, *Pparg*, and *Shbg* were down-regulated in PCOS-like mouse models (**Supplementary Figure 7B**). In addition, we analyzed the inter-chromosomal interactions between candidate genes and rs13405728 locus with Hi-C data. Although these candidate genes have been shown the association with PCOS, no single candidate gene showed inter-chromosomal interactions with rs13405728 locus (**Supplementary Figure 7C**). This may be attributed to the disease heterogeneity observed in PCOS (44). Since fat tissue is the target of insulin resistance and metabolic disorder in PCOS (45), we explored the expression levels of these candidate genes in adipose tissue of Macaca mulatta (macaque) among normal diet, testosterone treatment, western-style diet, and the combination of testosterone treatment and western-style diet groups from GSE124707. *IRS1* was up-regulated after testosterone treatment. Although these candidate genes showed slight expression differences compared to the normal diet group, no single gene showed statistical significance (**Supplementary Figure 8**).

The current data did not show an eQTL correlation at rs13405728 locus for *STON1* and *FSHR*. It is possible that the effects of the risk variants for *STON1* and *FSHR* were not validated in the PCOS cohort and hence not detected in this study. Although the data presented herein provided statistical differences between PCOS and normal controls, and the gene nearby the locus may be the potential candidates for PCOS, particularly concerning adipocyte metabolic and *CD4* immunological processes, further studies should be performed to determine the roles of the rs13405728 locus, *STON1*, and *FSHR* in the pathogenesis of PCOS.

In summary, 3D genomic interactions in primary ovary tissue identified the interaction genes at the rs13405728 locus as *STON1* and *FSHR*, which were highly expressed in PCOS patients. Further analysis showed the adipocyte metabolism roles of *STON1*, which was validated in the adipose tissue and ovaries of the fat-diet mice. In addition, immune system processes were enriched in PCOS, with *CD4* high expression in PCOS patients and PCOS-like models, which was consistent with the *CD4* immunological correlation of *FHSR* in PCOS patients and PCOS-like models. Furthermore, we found that *AR* was the common transcription factor for *STON1* and *FSHR* and positively correlated with *STON1* and *FSHR* expression in ovary tissues. Overall, we identified *STON1* and *FSHR* as potential targets of rs13405728 locus in adipocyte metabolism and immune processes in the pathogenesis of PCOS.

## Data Availability Statement

The original contributions presented in the study are included in the article/**Supplementary Material**. Further inquiries can be directed to the corresponding authors.

## Ethics Statement

The animal study was reviewed and approved by Ethics Committee of Shenzhen Peking University-The Hong Kong University of Science and Technology Medical Center.

## Author Contributions

C-hC performed the analysis of data, C-hC, YW, and X-rL performed the animal experiments, C-hC performed IHC of tissue. C-hC wrote the manuscript. J-qL, Q-jZ, S-rL, LG, and S-kY provided feedback. YS and XX designed the study and funding acquisition. All authors contributed to the article and approved the submitted version.

## Funding

This work was supported by the Research Team of Female Reproductive Health and Fertility Preservation (SZSM201612065) and the Shenzhen Science and Technology Innovation Committee (JCYJ20200109150429414 and JCYJ2017041217856582).

## Acknowledgments

We would like to acknowledge the platform of TCGA, GEO, GEPIA, GTEx, Metascape, 3Div, and 3D Genome Browser.

## Conflict of Interest

The authors declare that the research was conducted in the absence of any commercial or financial relationships that could be construed as a potential conflict of interest.
