# Supplementary material for: Three-Dimensional Genome Interactions Identify Potential Adipocyte Metabolism-Associated Gene STON1 and Immune-Correlated Gene FSHR at the rs13405728 Locus in Polycystic Ovary Syndrome
Source: Front Endocrinol (Lausanne). 2021 Jun 24;12:686054. doi: 10.3389/fendo.2021.686054 (PMC8264658; doi:10.3389/fendo.2021.686054)
Supplement: Supplementary file 1 [file DataSheet_1.docx]

**
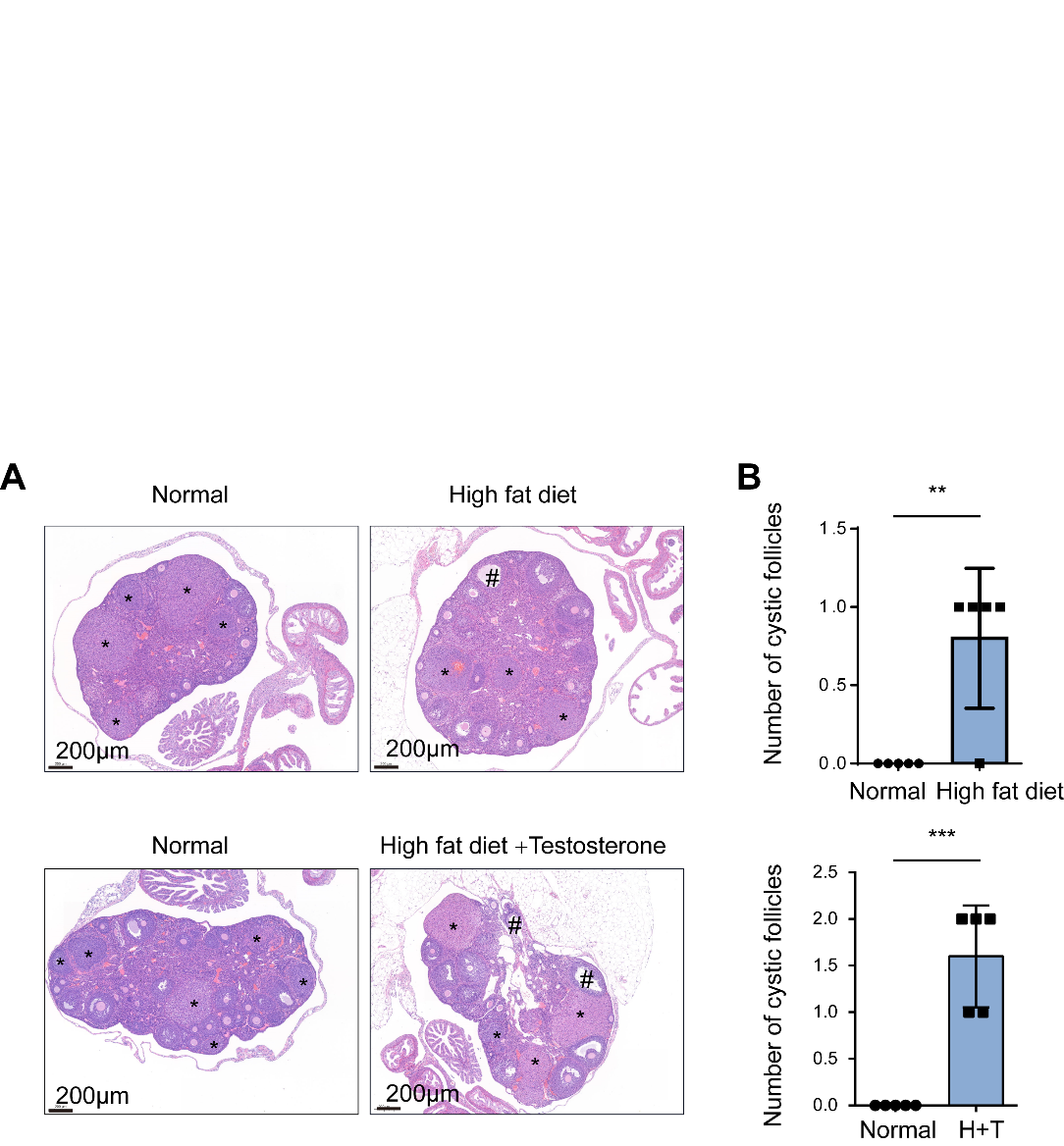
**

**Supplementary Figure 1: Results of PCOS-like models.** Hematoxylin and eosin (H&E) staining of representative ovaries (**A**) and quantitative analysis of cystic follicles (**B**). * represents corpus luteum, # represents the cystic follicle. P values were calculated by Student's t-test. * <0.05, **<0.001, ***<0.0001,


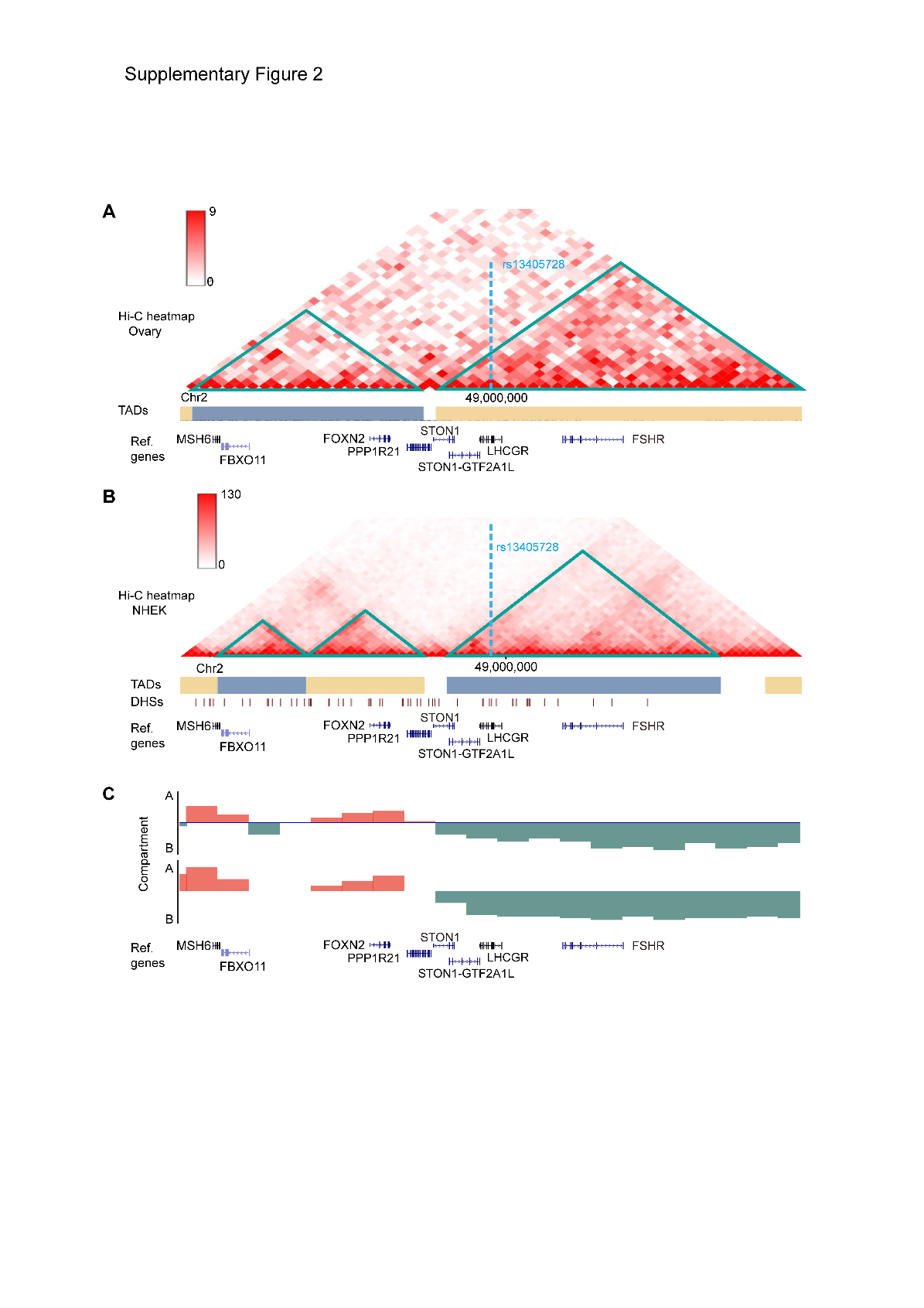


**Supplementary Figure 2: 3D genome analysis of tissues.** Hi-C heatmap of the ovary (**A**) and NHEK (**B**) in the region of the rs13405728 locus (Chr2: 48478159-49478159). (**C**) A/B compartment of normal cervical cells and cervical carcinoma in the he region of the rs13405728 locus (Chr2: 48478159-49478159).


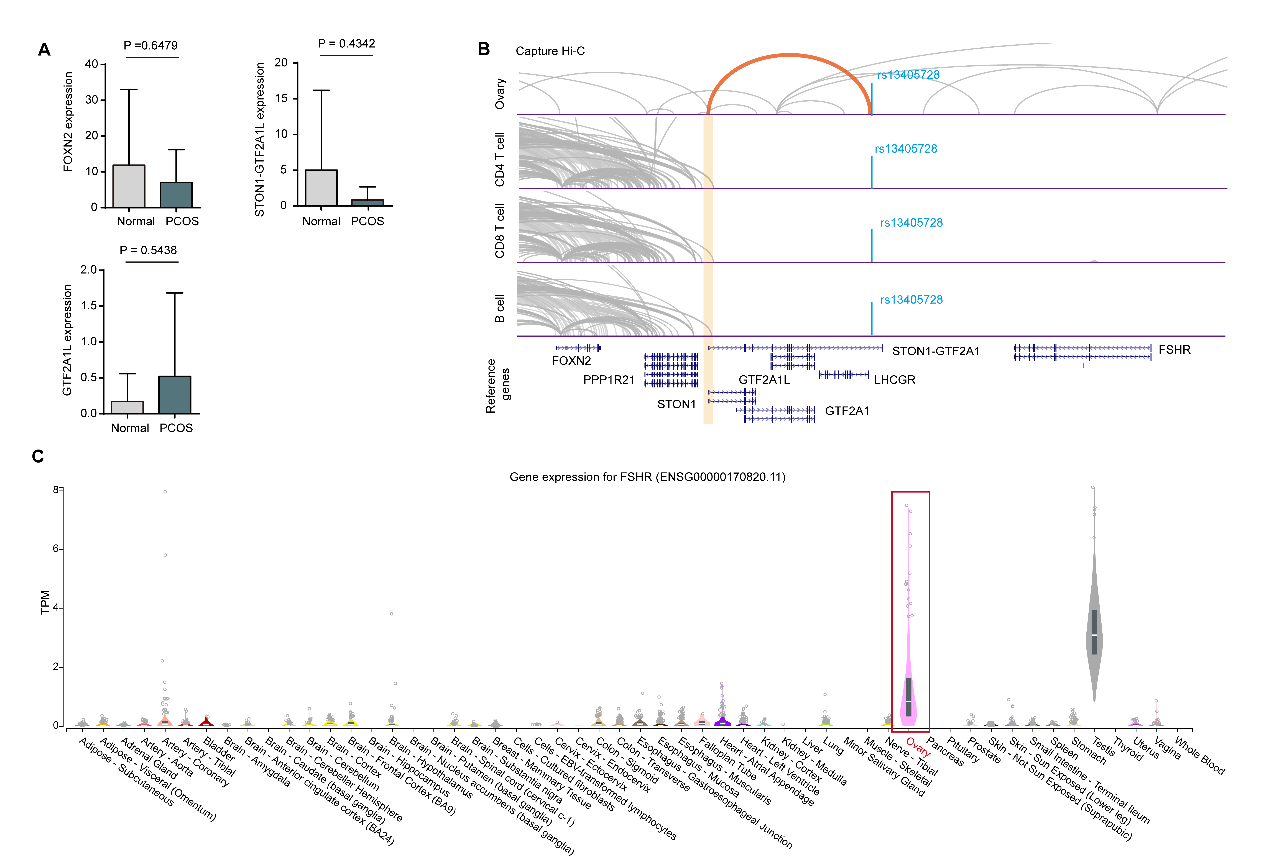


**Supplementary Figure 3: The expression of genes near the rs13405728 locus and Capture Hi-C analysis.** (**A**) The expression of FOXN2, STON1-GTF2A1L, and GTF2A1L in the GSE145461 dataset. (**B**) Capture Hi-C interactions of ovary tissue, CD4+ T cells, CD8+ T cells, B cells in the Chr2:48478158-49478158 region, the rs13405728 locus is indicated. (**C**) The expression of FSHR in multi-tissues of humans from the GTEx dataset. GTEx: Genotype-Tissue Expression.


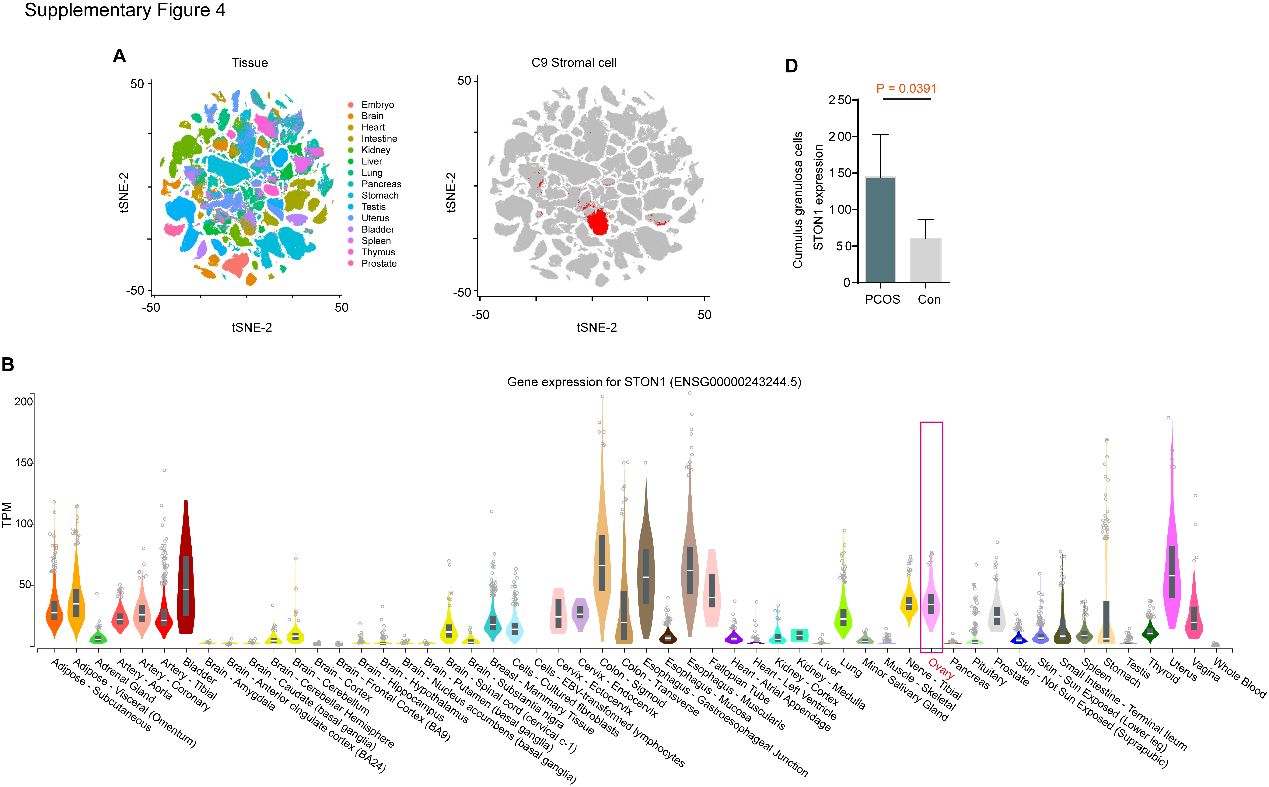


**Supplementary Figure 4: The expression patterns of STON1.** (**A**) Single-cell sequencing map of mouse tissues and the C9 cluster in the tSNE map. (**B**) The expression of STON1 in the PCOS patients and normal controls from the GSE155489 dataset. (**C**) The expression of STON1 in multi-tissues of humans from the GTEx dataset. GTEx: Genotype-Tissue Expression.


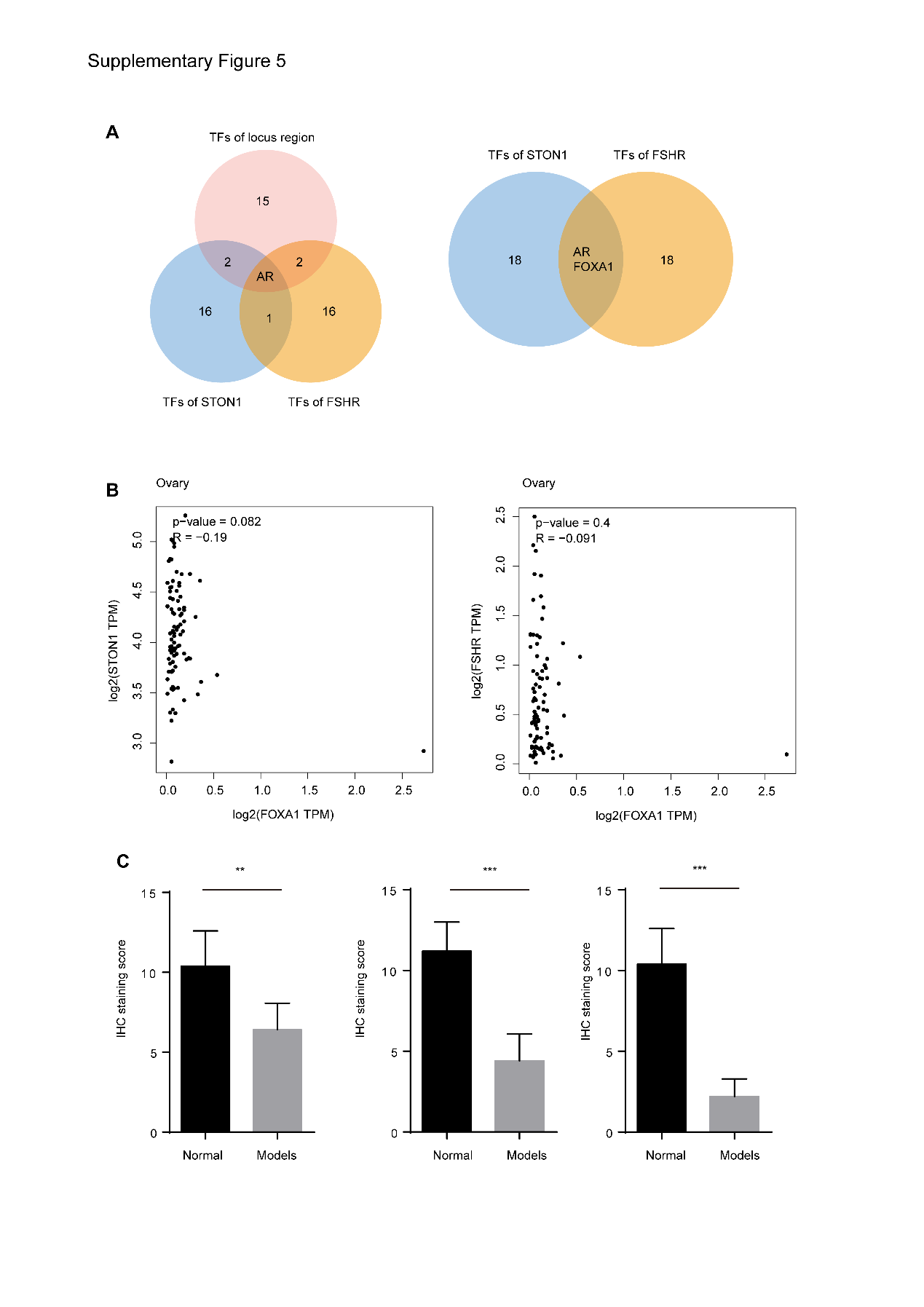


**Supplementary Figure 5: The transcrption factor analysis.** (**A**) Venn diagrams of TFs in the region of the rs13405728 locus (Chr2: 48478159-49478159) and gene regions of STON1 and FSHR. (**B**) Correlation analysis between the expression of FOXA1 and STON1, FOXA1 and FSHR in ovary tissues from GTEx dataset. (**C**) IHC staining scores of Ar, Ston1, and Fshr in the ovarian tissues from normal mice and PCOS-like models. P values were calculated by Student's t-test. * <0.05, **<0.001, ***<0.0001.


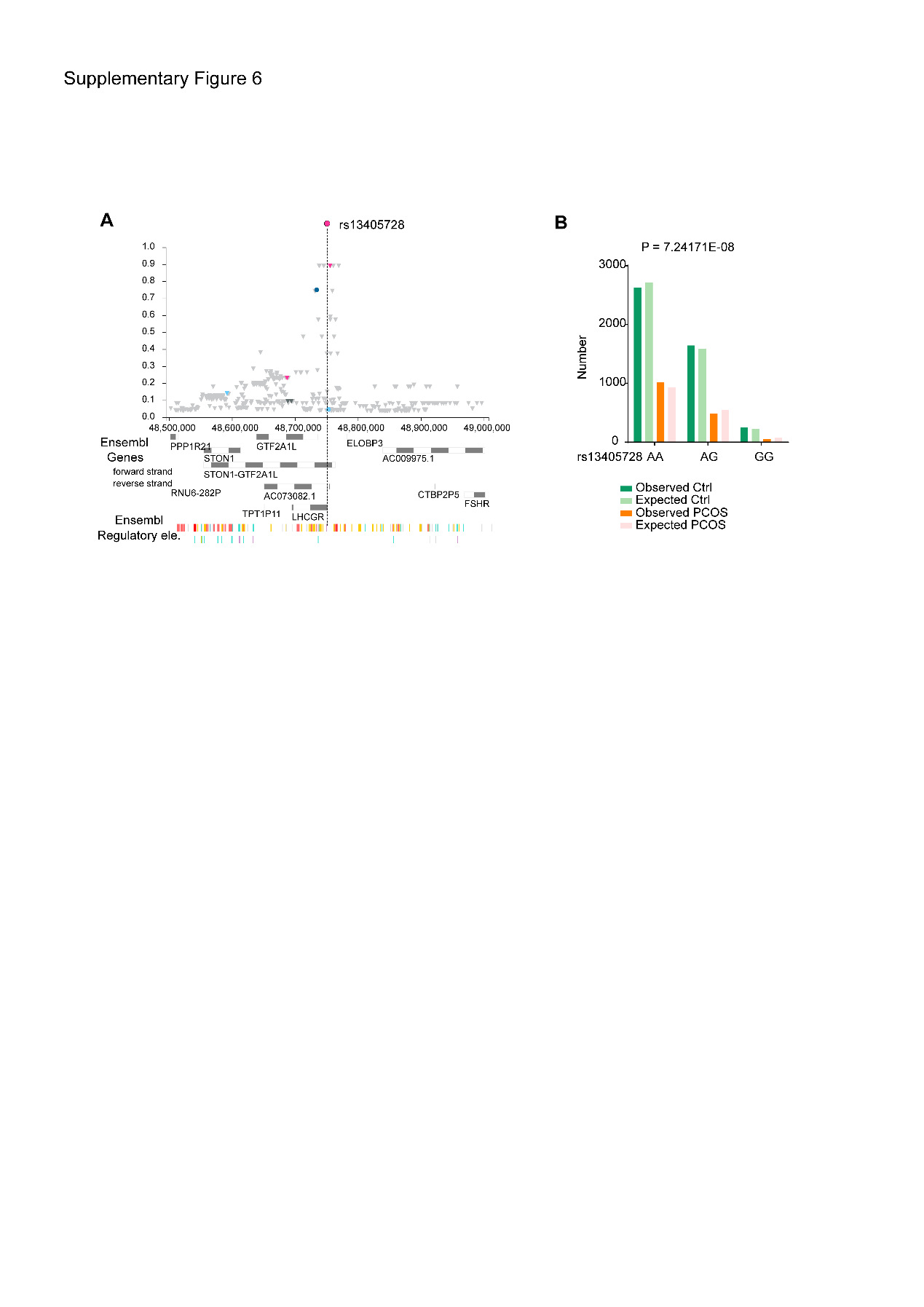


**Supplementary Figure 6: rs13405728 locus analysis.** (**A**) GWAS-associated SNP of rs13405728 locus from GWAS Catalog (https://www.ebi.ac.uk/gwas/). (**B**) Histogram of SNP in the rs13405728 locus from the groups of PCOS patients and normal controls.


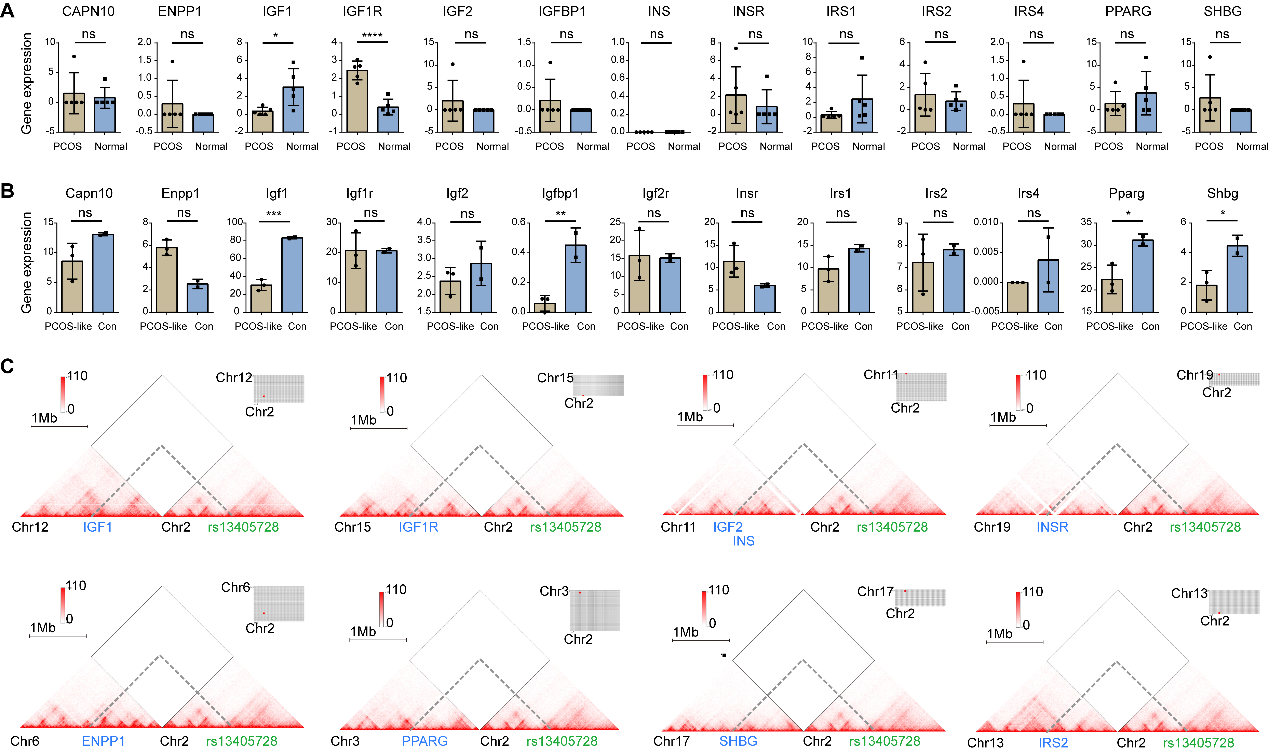


**Supplementary Figure 7: Candidate genes analysis related to insulin resistance in PCOS.** Gene expression levels in different groups from the GSE145461 dataset (**A**) and the GSE156895 dataset (**B**). (**C**) Inter-chromosomal interactions between candidate genes and rs13405728 locus with Hi-C data. The heatmaps were generated by an online tool (http://3dgenome.fsm.northwestern.edu/). P values were calculated by Student's t-test. * <0.05, **<0.001, ***<0.0001, ****<0.00001. ns: no significance.


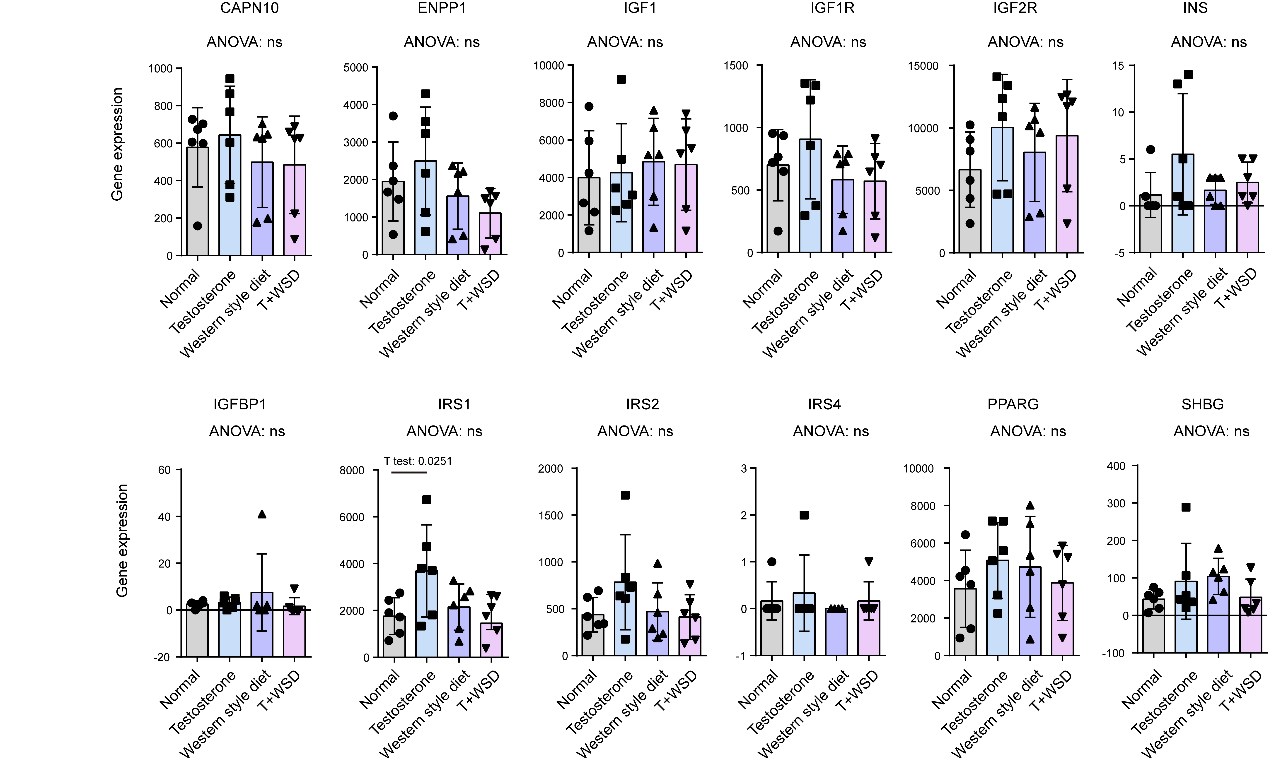


**Supplementary Figure 8: Gene expression levels in different groups from the GSE124707 dataset.** ns: no significance.
