# Supplementary material for: Three-Dimensional Genome Interactions Identify Potential Adipocyte Metabolism-Associated Gene STON1 and Immune-Correlated Gene FSHR at the rs13405728 Locus in Polycystic Ovary Syndrome
Source: Front Endocrinol (Lausanne). 2021 Jun 24;12:686054. doi: 10.3389/fendo.2021.686054 (PMC8264658; doi:10.3389/fendo.2021.686054)
Supplement: Supplementary file 2 [file DataSheet_2.pdf]

Supplementary Table 1: The expression of FSHR in the single-cell sequencing data of mouse ovary.

Cluster	Cell annotation	Fshr expression
Cluster1	Cumulus cell_Car14 high	0.5804
Cluster2	Small luteal cell	0
Cluster3	Luteal cell	0.1236
Cluster4	Stroma cell	0.0919
Cluster5	Thecal cell	0.0197
Cluster6	Cumulus cell_Ube2c high	1.103
Cluster7	Cumulus cell_Nupr1 high	0.1156
Cluster8	Granulosa cell_Kctd14 high	0.2412
Cluster9	Granulosa cell_Inhba high	0.5327
Cluster10	Large luteal cell	0.1068
Cluster11	Endothelial cell	0.0606
Cluster12	Macrophage_Lyz2 high	0
Cluster13	Epithelial cell	0
Cluster14	Marcrophage_Cd74 high	0

Supplementary Table 2: Dataset information of PCOS or PCOS-like models.

GEO accession	Tissue	Experimental group	Experiment number	Control	Control number	Species
GSE145461	Follicle fluid	PCOS patients	5	Normal people	5	Homo sapiens
GSE156895	Ovarian samples	Prenatally androgenized (PNA) mice	3	Normal control	2	Mus musculus
GSE114419	Luteinized granulosa cells	PCOS patients	3	Normal people	3	Homo sapiens
GSE138518	Ovarian granulosa cells	PCOS patients	3	Normal people	3	Homo sapiens
GSE8157	Skeletal muscle	PCOS patients	10	ns	ns	Homo sapiens
GSE124707	Adipose tissue	T/T+WSD/WSD	6//6/6	Normal control	6	Macaca mulatta
GSE135917	Adipose tissue	PCOS patients	8	Normal people	7	Homo sapiens

Supplementary Table 3: Phenotypes of PCOS patients

	GSE145461	GSE114419	GSE138518	GSE8157	P-value[a]
Race	Chinese	Chinese	Han Chinese	Caucasian	/
PCOS patients	5	3	3	10	/
Age	29.6 ± 1.15	28 ± 1	29.3 ± 3.89	30.3 ± 2.1	0.4049
BMI (Kg/m2)	22.06 ± 0.79	21.0 ± 1.4	24.82 ± 3.36	33.2 ± 0.9	<0.0001
H(IU/L)	11.91 ± 2.16	8.1 ± 2.1	7.76 ± 3.88	/	0.1059
FSH(IU/L)	5.21 ± 0.92	5.3 ± 0.4	5.01 ± 1.68	/	0.9445
Testosterone(ng/dL)	33 ± 5	69.8 ± 13.5	169 ± 94	/	0.0135
AMH (ng/mL)	11.9 ± 3.9	/	/	/	/

[a] P values were calculated by ANOVA test

Supplementary Table 4: Hi-C interactions in the ovary tissue.

Sample	Bin	Distance	Bias-removec	Distance nor	Enhancer	GWAS SNP	Gene Name
Ovary	chr2:49175000-49180000	200000.00	5.11	2.24	None	rs7591064	-
Ovary	chr2:49200000-49205000	225000.00	3.64	1.65	None	rs2268363	-
Ovary	chr2:48795000-48800000	180000.00	5.43	2.32	None	-	STON1-GTF2A1L
Ovary	chr2:48805000-48810000	170000.00	4.60	1.93	None	-	STON1-GTF2A1L
Ovary	chr2:48755000-48760000	220000.00	0.58	0.26	None	-	STON1,STON1-GTF2A1L
Ovary	chr2:48840000-48845000	135000.00	1.00	0.39	None	-	GTF2A1L
Ovary	chr2:49380000-49385000	405000.00	3.75	1.95	None	-	FSHR
Ovary	chr2:48540000-48545000	435000.00	1.03	0.55	None	-	FOXN2
Ovary	chr2:48740000-48745000	235000.00	4.12	1.89	Enhancer	-	-
Ovary	chr2:48530000-48535000	445000.00	0.12	0.07	None	-	-
Ovary	chr2:48535000-48540000	440000.00	0.55	0.29	None	-	-
Ovary	chr2:48545000-48550000	430000.00	1.10	0.58	None	-	-
Ovary	chr2:48550000-48555000	425000.00	1.03	0.54	None	-	-
Ovary	chr2:48555000-48560000	420000.00	0.55	0.29	None	-	-
Ovary	chr2:48560000-48565000	415000.00	0.12	0.06	None	-	-
Ovary	chr2:48590000-48595000	385000.00	1.66	0.85	None	-	-
Ovary	chr2:48595000-48600000	380000.00	7.43	3.79	None	-	-
Ovary	chr2:48600000-48605000	375000.00	12.25	6.22	None	-	-
Ovary	chr2:48605000-48610000	370000.00	7.43	3.76	None	-	-
Ovary	chr2:48610000-48615000	365000.00	1.66	0.84	None	-	-
Ovary	chr2:48630000-48635000	345000.00	0.64	0.32	None	-	-
Ovary	chr2:48635000-48640000	340000.00	2.88	1.43	None	-	-
Ovary	chr2:48640000-48645000	335000.00	4.84	2.39	None	-	-
Ovary	chr2:48645000-48650000	330000.00	3.27	1.61	None	-	-
Ovary	chr2:48650000-48655000	325000.00	1.29	0.63	None	-	-
Ovary	chr2:48655000-48660000	320000.00	0.39	0.19	None	-	-
Ovary	chr2:48660000-48665000	315000.00	0.09	0.04	None	-	-
Ovary	chr2:48725000-48730000	250000.00	0.92	0.43	None	-	-
Ovary	chr2:48730000-48735000	245000.00	4.12	1.91	None	-	-
Ovary	chr2:48735000-48740000	240000.00	6.80	3.13	None	-	-
Ovary	chr2:48745000-48750000	230000.00	0.92	0.42	None	-	-
Ovary	chr2:48760000-48765000	215000.00	2.62	1.17	None	-	-
Ovary	chr2:48765000-48770000	210000.00	5.28	2.35	None	-	-
Ovary	chr2:48770000-48775000	205000.00	7.07	3.12	None	-	-
Ovary	chr2:48775000-48780000	200000.00	8.29	3.64	None	-	-
Ovary	chr2:48780000-48785000	195000.00	5.28	2.30	None	-	-
Ovary	chr2:48785000-48790000	190000.00	1.68	0.73	None	-	-
Ovary	chr2:48790000-48795000	185000.00	1.71	0.74	None	-	-
Ovary	chr2:48800000-48805000	175000.00	7.96	3.37	None	-	-
Ovary	chr2:48810000-48815000	165000.00	1.00	0.42	None	-	-
Ovary	chr2:48820000-48825000	155000.00	1.00	0.41	None	-	-
Ovary	chr2:48825000-48830000	150000.00	4.47	1.81	None	-	-
Ovary	chr2:48830000-48835000	145000.00	7.37	2.94	None	-	-
Ovary	chr2:48835000-48840000	140000.00	4.47	1.77	None	-	-
Ovary	chr2:48860000-48865000	115000.00	0.66	0.24	None	-	-
Ovary	chr2:48865000-48870000	110000.00	3.62	1.30	None	-	-
Ovary	chr2:48870000-48875000	105000.00	7.85	2.76	None	-	-
Ovary	chr2:48875000-48880000	100000.00	7.85	2.70	None	-	-
Ovary	chr2:48880000-48885000	95000.00	3.62	1.21	None	-	-
Ovary	chr2:48885000-48890000	90000.00	0.66	0.21	None	-	-
Ovary	chr2:48920000-48925000	55000.00	0.11	0.03	None	-	-
Ovary	chr2:48925000-48930000	50000.00	0.52	0.11	None	-	-
Ovary	chr2:48930000-48935000	45000.00	0.85	0.17	None	-	-
Ovary	chr2:48935000-48940000	40000.00	0.52	0.10	None	-	-
Ovary	chr2:48940000-48945000	35000.00	1.00	0.19	None	-	-
Ovary	chr2:48945000-48950000	30000.00	6.26	1.11	None	-	-
Ovary	chr2:48950000-48955000	25000.00	18.28	3.08	None	-	-
Ovary	chr2:48955000-48960000	20000.00	27.27	4.40	None	-	-
Ovary	chr2:48995000-49000000	20000.00	19.23	3.10	None	-	-
Ovary	chr2:49000000-49005000	25000.00	9.21	1.55	None	-	-
Ovary	chr2:49005000-49010000	30000.00	1.77	0.31	None	-	-
Ovary	chr2:49020000-49025000	45000.00	1.66	0.34	None	-	-
Ovary	chr2:49025000-49030000	50000.00	7.62	1.65	None	-	-
Ovary	chr2:49030000-49035000	55000.00	13.01	2.99	None	-	-

Ovary	chr2:49035000 -49040000	60000 .00	9.08	2.22	None	-	-
Ovary	chr2:49040000 -49045000	65000 .00	4.37	1.13	None	-	-
Ovary	chr2:49045000 -49050000	70000 .00	3.44	0.95	None	-	-
Ovary	chr2:49050000 -49055000	75000 .00	2.43	0.71	None	-	-
Ovary	chr2:49055000 -49060000	80000 .00	2.43	0.74	None	-	-
Ovary	chr2:49060000 -49065000	85000 .00	3.27	1.03	None	-	-
Ovary	chr2:49065000 -49070000	90000 .00	1.99	0.64	None	-	-
Ovary	chr2:49070000 -49075000	95000 .00	1.25	0.42	None	-	-
Ovary	chr2:49075000 -49080000	100000 .00	3.64	1.25	None	-	-
Ovary	chr2:49080000 -49085000	105000 .00	5.99	2.11	None	-	-
Ovary	chr2:49085000 -49090000	110000 .00	3.64	1.31	None	-	-
Ovary	chr2:49090000 -49095000	115000 .00	1.30	0.48	None	-	-
Ovary	chr2:49095000 -49100000	120000 .00	2.21	0.82	None	-	-
Ovary	chr2:49100000 -49105000	125000 .00	3.64	1.38	None	-	-
Ovary	chr2:49105000 -49110000	130000 .00	2.31	0.89	None	-	-
Ovary	chr2:49110000 -49115000	135000 .00	0.98	0.38	None	-	-
Ovary	chr2:49115000 -49120000	140000 .00	0.89	0.35	None	-	-
Ovary	chr2:49120000 -49125000	145000 .00	1.47	0.59	None	-	-
Ovary	chr2:49125000 -49130000	150000 .00	3.52	1.42	None	-	-
Ovary	chr2:49130000 -49135000	155000 .00	5.83	2.38	None	-	-
Ovary	chr2:49135000 -49140000	160000 .00	6.14	2.53	None	-	-
Ovary	chr2:49140000 -49145000	165000 .00	5.47	2.27	None	-	-
Ovary	chr2:49145000 -49150000	170000 .00	3.41	1.43	None	-	-
Ovary	chr2:49150000 -49155000	175000 .00	3.33	1.41	None	-	-
Ovary	chr2:49155000 -49160000	180000 .00	4.58	1.95	None	-	-
Ovary	chr2:49160000 -49165000	185000 .00	3.13	1.35	None	-	-
Ovary	chr2:49165000 -49170000	190000 .00	1.96	0.85	None	-	-
Ovary	chr2:49170000 -49175000	195000 .00	3.45	1.51	None	-	-
Ovary	chr2:49180000 -49185000	205000 .00	3.04	1.34	None	-	-
Ovary	chr2:49185000 -49190000	210000 .00	1.36	0.60	None	-	-
Ovary	chr2:49190000 -49195000	215000 .00	3.12	1.40	None	-	-
Ovary	chr2:49195000 -49200000	220000 .00	5.38	2.42	None	-	-
Ovary	chr2:49205000 -49210000	230000 .00	1.04	0.47	None	-	-
Ovary	chr2:49210000 -49215000	235000 .00	0.08	0.04	None	-	-
Ovary	chr2:49250000 -49255000	275000 .00	0.12	0.06	None	-	-
Ovary	chr2:49255000 -49260000	280000 .00	0.53	0.26	None	-	-
Ovary	chr2:49260000 -49265000	285000 .00	0.88	0.42	None	-	-
Ovary	chr2:49265000 -49270000	290000 .00	0.53	0.26	None	-	-
Ovary	chr2:49270000 -49275000	295000 .00	0.12	0.06	None	-	-
Ovary	chr2:49280000 -49285000	305000 .00	0.17	0.08	None	-	-
Ovary	chr2:49285000 -49290000	310000 .00	0.91	0.44	None	-	-
Ovary	chr2:49290000 -49295000	315000 .00	1.96	0.96	None	-	-
Ovary	chr2:49295000 -49300000	320000 .00	2.13	1.04	None	-	-
Ovary	chr2:49300000 -49305000	325000 .00	1.65	0.81	None	-	-
Ovary	chr2:49305000 -49310000	330000 .00	1.39	0.68	None	-	-
Ovary	chr2:49310000 -49315000	335000 .00	0.74	0.37	None	-	-
Ovary	chr2:49315000 -49320000	340000 .00	0.17	0.08	None	-	-
Ovary	chr2:49330000 -49335000	355000 .00	0.67	0.34	None	-	-
Ovary	chr2:49335000 -49340000	360000 .00	3.02	1.52	None	-	-
Ovary	chr2:49340000 -49345000	365000 .00	5.07	2.56	None	-	-
Ovary	chr2:49345000 -49350000	370000 .00	3.43	1.74	None	-	-
Ovary	chr2:49350000 -49355000	375000 .00	1.35	0.68	None	-	-
Ovary	chr2:49355000 -49360000	380000 .00	0.41	0.21	None	-	-
Ovary	chr2:49360000 -49365000	385000 .00	0.09	0.05	None	-	-
Ovary	chr2:49365000 -49370000	390000 .00	0.69	0.35	None	-	-
Ovary	chr2:49370000 -49375000	395000 .00	3.07	1.58	None	-	-
Ovary	chr2:49375000 -49380000	400000 .00	5.06	2.62	None	-	-
Ovary	chr2:49385000 -49390000	410000 .00	3.75	1.96	None	-	-
Ovary	chr2:49390000 -49395000	415000 .00	5.75	3.01	None	-	-
Ovary	chr2:49395000 -49400000	420000 .00	6.84	3.60	None	-	-
Ovary	chr2:49400000 -49405000	425000 .00	8.86	4.67	None	-	-
Ovary	chr2:49405000 -49410000	430000 .00	8.17	4.32	None	-	-
Ovary	chr2:49410000 -49415000	435000 .00	3.77	2.00	None	-	-
Ovary	chr2:49415000 -49420000	440000 .00	0.69	0.37	None	-	-
Ovary	chr2:49440000 -49445000	465000 .00	0.54	0.29	None	-	-
Ovary	chr2:49445000 -49450000	470000 .00	2.42	1.31	None	-	-
Ovary	chr2:49450000 -49455000	475000 .00	3.99	2.16	None	-	-
Ovary	chr2:49455000 -49460000	480000 .00	2.42	1.31	None	-	-
Ovary	chr2:49460000 -49465000	485000 .00	0.54	0.29	None	-	-

Supplementary Table 5: TFs of the region at the rs13405728 locus.

GSM_ID	Factor	Biosource	All_peak_number	Overlap peak num.	Overlap_ratio
GSM2472121	SMARCC1	SK-N-MC;Brain	1470	8	0.005442177
GSM2472120	SMARCA2	SK-N-MC;Brain	1716	5	0.002913753
GSM2419822	RELA	Detroit 562;Detroit 562 cells	2252	6	0.002664298
GSM2419827	RELA	Detroit 562;Detroit 562 cells	2292	6	0.002617801
GSM1505799	TRIM28	HUES64;Embryonic Stem Cell;Embryo	1248	3	0.002403846
GSM1505745	SMAD1	HUES64;Embryonic Stem Cell;Embryo	1830	4	0.002185792
GSM1505639	FOXA2	HUES64;Embryonic Stem Cell;Embryo	2005	4	0.001995012
GSM2401446	FOXA2	BJ;Fibroblast;Skin	9843	17	0.001727116
GSM2817662	GATA2	Embryonic Stem Cell	3245	5	0.001540832
GSM2474870	SIRT1	K562;Leukemia	3504	5	0.001426941
GSM2466569	ZNF480	293T	1508	2	0.00132626
GSM1505763	SOX17	HUES64;Embryonic Stem Cell;Embryo	1518	2	0.001317523
GSM2817661	GATA3	Embryonic Stem Cell	2281	3	0.001315213
ENCSR081WLS_2	RAD51	HepG2;Epithelium;Liver	2282	3	0.001314636
GSM2670862	ESR1	MCF-7;Epithelium;Breast	6606	8	0.00121102
GSM353651	AR	Epithelium;Prostate	3379	4	0.001183782
GSM1197322	GATA6	LS174T;Epithelium;Colon	6938	8	0.00115307
GSM1682262	AGO2	K562;Erythroblast;Bone Marrow	1767	2	0.001131862
GSM881710	POU5F1	NCCIT;Embryo	2662	3	0.001126972
GSM1197321	GATA6	LS174T;Epithelium;Colon	8028	9	0.001121076
GSM837987	TCF12	CCRF-CEM;T Lymphocyte;Blood	1845	2	0.001084011
GSM2612451	ERF	VCaP;Epithelium;Prostate	3698	4	0.001081666
GSM2026881	ZNF331	HEK293;Epithelium;Embryonic Kidney	1907	2	0.001048768
GSM717404	EZH2	VCaP;Epithelium;Prostate	1952	2	0.00102459
GSM1076112	ESR1	NCI-H660;Epithelium;Prostate	5017	5	0.000996612
GSM2026874	ZBTB26	HEK293;Epithelium;Embryonic Kidney	2015	2	0.000992556
GSM1706694	HDAC2	hESC;Embryonic Stem Cell	1015	1	0.000985222
GSM1410771	RUNX1	VCaP;Epithelium;Prostate	4090	4	0.000977995
GSM1602666	GATA3	KELLY	21516	21	0.000976018
GSM2472122	SMARCC1	SK-N-MC;Brain	1028	1	0.000972763
GSM1505813	HAND1	HUES64;Embryonic Stem Cell;Embryo	1038	1	0.000963391
GSM2797142	NR3C1	Breast	1045	1	0.000956938
GSM1295632	TCF7	DLD-1;Epithelium;Colon	1050	1	0.000952381
GSM2817660	GATA3	Embryonic Stem Cell	3201	3	0.000937207
GSM1505663	GATA6	HUES64;Embryonic Stem Cell;Embryo	7517	7	0.000931223
GSM1505724	POU5F1	HUES64;Embryonic Stem Cell;Embryo	1077	1	0.000928505
GSM1517537	FLI1	SK-N-MC;Brain	3285	3	0.000913242
ENCSR772EEN_2	RELA	K562;Erythroblast;Bone Marrow	1098	1	0.000910747
GSM2817663	GATA2	Embryonic Stem Cell	5564	5	0.000898634
GSM831033	KDM5B	H1;Embryonic Stem Cell;Embryo	1131	1	0.000884173
GSM1003522	EZH2	DND-41;T Lymphocyte;Blood	1141	1	0.000876424
ENCSR772EEN_1	RELA	K562;Erythroblast;Bone Marrow	1150	1	0.000869565
GSM545809	TP53	U2OS;Bone	1152	1	0.000868056
GSM545203	POU5F1	H9;Embryonic Stem Cell;Embryo	5816	5	0.000859697
GSM1410776	FOXA1	VCaP;Epithelium;Prostate	4665	4	0.000857449
GSM908065	CTCF	Proerythroblast;Bone Marrow	1183	1	0.000845309
GSM1056913	H2AZ	B Lymphocyte;Blood	5978	5	0.0008364
GSM2026809	ZNF528	HEK293;Epithelium;Embryonic Kidney	1198	1	0.000834725
GSM1517546	FLI1	SK-N-MC;Brain	3594	3	0.000834725
GSM2296271	TP53	HCT-116;colorectal adenocarcinoma;HCT116	1199	1	0.000834028
GSM545810	TP53	U2OS;Bone	1207	1	0.0008285
GSM1197320	GATA6	LS174T;Epithelium;Colon	10944	9	0.000822368
GSM1505631	EOMES	HUES64;Embryonic Stem Cell;Embryo	1218	1	0.000821018
GSM2664370	GATA3	CLB-Ga;Neuroblastoma cell	11009	9	0.000817513
GSM1139041	SOX2	H9;Embryonic Stem Cell;Embryo	2448	2	0.000816993
GSM1071289	AR	Epithelium;Prostate	2453	2	0.000815328
GSM1527528	PGR	Stromal Cell;Endometrium	6143	5	0.000813935
GSM1934417	POU3F2	Neural Progenitor Cell	1229	1	0.00081367
GSM1505742	SMAD1	HUES64;Embryonic Stem Cell;Embryo	2462	2	0.000812348
GSM1537616	POU5F1	NT2-D1;Embryo	1234	1	0.000810373
GSM1071287	AR	Epithelium;Prostate	4937	4	0.000810209
GSM2101199	FOXM1	VAL-3;Embryonic Stem Cell	2469	2	0.000810045
GSM2026880	ZNF331	HEK293;Epithelium;Embryonic Kidney	2469	2	0.000810045

GSM1505712	OTX2	HUES64;Embryonic Stem Cell;Embryo	3709	3	0.000808843
GSM1193656	ERG	VCaP;Epithelium;Prostate	1242	1	0.000805153
GSM1505714	PAX6	HUES64;Embryonic Stem Cell;Embryo	1245	1	0.000803213
GSM2719766	ARID1A	HCT-116;HCT116	1248	1	0.000801282
GSM1892296	SMN1	HEK293;Epithelium;Embryonic Kidney	1263	1	0.000791766
GSM1835882	SMARCA4	BT-16;Brain	3803	3	0.000788851
GSM1579343	LEF1	Embryonic Stem Cell	1269	1	0.000788022
GSM2406725	AHRR	MCF-7;Epithelium;Breast	1278	1	0.000782473
GSM1294890	TP53	IMR90;Fibroblast;Lung	1280	1	0.00078125
GSM2393829	AR	LNCaP;Epithelium;Prostate	2563	2	0.000780336
GSM1405131	ERCC6	Fibroblast;Skin	1301	1	0.00076864
GSM1532287	FOXP1	OF4155;Neural Progenitor Cell	1302	1	0.000768049
GSM1544111	MBD2	HMEC-hTERT	1322	1	0.00075643
GSM2040030	SMC1A	HepG2;hepatocellular carcinoma	13237	10	0.000755458
GSM2154996	GATA3	MCF-7;Breast	3980	3	0.000753769
GSM2058886	FOXA1	VCaP;Epithelium;Prostate	3981	3	0.00075358
GSM981235	TP53	H9;Embryonic Stem Cell;Embryo	1337	1	0.000747943
GSM2398965	SP140	Macrophage	2682	2	0.000745712
GSM2514497	SETDB1	HeLa;Epithelium;Cervix	2690	2	0.000743494
GSM791414	CDX2	LS180;Epithelium;Colon	43136	32	0.00074184
GSM2612449	ERF	VCaP;Epithelium;Prostate	4066	3	0.000737826
GSM2086310	ERG	VCaP;Epithelium;Prostate	4078	3	0.000735655
GSM1505700	NANOG	HUES64;Embryonic Stem Cell;Embryo	12271	9	0.000733437
GSM2232927	TEAD4	BE2-C;Neuroblastoma cell;Bone Marrow	46436	34	0.000732191
GSM2797128	AR	Breast	2750	2	0.000727273
GSM1505660	GATA6	HUES64;Embryonic Stem Cell;Embryo	4150	3	0.000722892
GSM1982476	HNF1A	Epithelium;Epididymis	11106	8	0.000720331
GSM1607527	NR3C1	U2OS;Bone	5554	4	0.000720202
GSM1358397	AR	Prostate	8375	6	0.000716418
GSM2257672	HIF1A	U2OS;Bone	6983	5	0.000716025
GSM2439240	CUX1	K562;Leukemia Cell	2809	2	0.000711997
GSM2664369	PHOX2B	CLB-Ga;Neuroblastoma cell	40894	29	0.00070915
GSM1035433	SUMO1	WI-38;Fibroblast;Lung	1412	1	0.000708215
GSM945571	POLR2A	Raji;B Lymphocyte;Blood	4240	3	0.000707547
GSM1174714	CHD5	SH-SY5Y;Bone Marrow	1415	1	0.000706714
GSM1517544	FLI1	SK-N-MC;Brain	4253	3	0.000705384
GSM1003619	ZNF274	H1;Embryonic Stem Cell;Embryo	1419	1	0.000704722
GSM798390	ESR1	Epithelium;Mammary Gland	2839	2	0.000704473
GSM1631484	TOP2B	MCF-7;Epithelium;Breast	1425	1	0.000701754
GSM1071284	AR	Epithelium;Prostate	5727	4	0.000698446
GSM2480811	SMARCA4	LNCaP;Epithelium;Prostate	2867	2	0.000697593
GSM1198994	SOX2	ReN-VM;Neural Stem Cell	10037	7	0.00069742
GSM1410773	AR	VCaP;Epithelium;Prostate	1442	1	0.000693481
GSM881712	POU5F1	NCCIT;Embryo	14429	10	0.000693049
GSM2188513	BRD4	L3.6;Pancreas	8661	6	0.000692761
GSM830997	HDAC6	K562;Erythroblast;Bone Marrow	1450	1	0.000689655
GSM2574779	NFIA	HepG2;Epithelium;Liver	2925	2	0.000683761
GSM986071	GATA3	MCF-7;Epithelium;Breast	4389	3	0.000683527
GSM2817670	TFAP2C	H9;Embryonic Stem Cell;Embryo	8810	6	0.000681044
GSM775364	AR	LNCaP;Epithelium;Prostate	1471	1	0.00067981
GSM1505711	OTX2	HUES64;Embryonic Stem Cell;Embryo	2953	2	0.000677277
GSM1701828	SOX2	Neuronal Progenitor cell	63587	43	0.000676239
GSM1505733	SALL4	HUES64;Embryonic Stem Cell;Embryo	1481	1	0.000675219
GSM1922948	RBPJ	Neurosphere	4451	3	0.000674006
ENCSR081WLS_1	RAD51	HepG2;Epithelium;Liver	5959	4	0.000671254
GSM803396	BCL11A	H1;Embryonic Stem Cell;Embryo	5974	4	0.000669568
GSM1842793	PRDM14	NCCIT;Embryonal carcinoma cell	1503	1	0.000665336
GSM1527529	PGR	Stromal Cell;Endometrium	12076	8	0.000662471
GSM1358396	AR	Prostate	22747	15	0.000659428
GSM791413	CDX2	LS180;Epithelium;Colon	30415	20	0.00065757
GSM468204	RXRG	Promyelocytic cell;Bone Marrow	6103	4	0.000655415
GSM1071294	AR	Epithelium;Prostate	6107	4	0.000654986
GSM2466620	ZNF627	293T	1528	1	0.00065445
GSM1705268	POU5F1	Embryonic Stem Cell;Embryo	15300	10	0.000653595

GSM869307	FOXO3	DL23;Epithelium;Colon	6140	4	0.000651466
GSM1375207	H3F3B	Spermatid	3076	2	0.000650195
GSM1555572	SUMO2	VCaP;Epithelium;Prostate	4630	3	0.000647948
GSM1034504	GRHL3	NHEK;Keratinocyte;Skin	10866	7	0.000644211
GSM1505645	GATA4	HUES64;Embryonic Stem Cell;Embryo	9322	6	0.000643639
GSM801053	CREB1	H3396;Epithelium;Mammary Gland	4692	3	0.000639386
GSM1607526	NR3C1	U2OS;Bone	35988	23	0.000639102
GSM1139038	SOX2	HCC95;Epithelium;Lung	3143	2	0.000636335
GSM1643797	SOX2	Neural Progenitor Cell	3165	2	0.000631912
GSM1076111	ESR1	VCaP;Epithelium;Prostate	1583	1	0.000631712
GSM2550188	EP300	Stem cell	6334	4	0.000631512
GSM2430681	FOXA2	BJ;Fibroblast;Skin	1585	1	0.000630915
ENCSR635GTR_1	TEAD2	K562;Erythroblast;Bone Marrow	3173	2	0.000630318
GSM1622630	PIAS1	VCaP;Epithelium;Prostate	1591	1	0.000628536
GSM1427074	EZH2	Proerythroblast;Fetal Liver	1593	1	0.000627746
GSM2635693	TEAD4	H1;Embryonic Stem Cell;Embryo	6390	4	0.000625978
GSM1505739	SMAD1	HUES64;Embryonic Stem Cell;Embryo	1598	1	0.000625782
GSM1071291	AR	Epithelium;Prostate	3204	2	0.00062422
GSM1464012	FOXM1	HEK293;Epithelium;Embryonic Kidney	1636	1	0.000611247
GSM2612457	ERG	VCaP;Epithelium;Prostate	9868	6	0.000608026
GSM1358395	AR	Prostate	8229	5	0.000607607
GSM2466639	ZNF707	293T	3326	2	0.000601323
GSM1888550	RUNX1	TF-1;Erythroblast;Bone Marrow	1663	1	0.000601323
GSM791404	VDR	LS180;Epithelium;Colon	5006	3	0.000599281
GSM1825754	T	T Lymphocyte	1683	1	0.000594177
GSM448026	BTAF1	HeLa;Epithelium;Cervix	3367	2	0.000594001
GSM634629	PPARG	SGBS;Adipocyte;Adipose	6753	4	0.000592329
GSM2817667	TFAP2A	Embryonic Stem Cell	25406	15	0.000590412
GSM1479212	AUTS2	293T-Rex	1697	1	0.000589275
GSM838668	BMI1	Neural Progenitor Cell	8541	5	0.000585412
GSM699986	E2F1	MCF-7;Epithelium;Breast	1717	1	0.000582411
GSM2144899	POLR2A	Nalm6;Leukemia Cell	1721	1	0.000581058
GSM848789	HOXC9	BE2-C;Neuroblast;Brain	36212	21	0.000579918
GSM1071286	AR	Epithelium;Prostate	5174	3	0.000579822
GSM2061657	ESR1	MCF-7;Breast	20698	12	0.000579766
ENCSR178NTX_2	CUX1	K562;Erythroblast;Bone Marrow	3450	2	0.00057971
GSM1515528	AR	R1-D567;Epithelium;Prostate	1733	1	0.000577034
GSM2280027	TBX5	Cardiomyocyte	8693	5	0.000575175
GSM700354	TRIM28	HEK293;Epithelium;Embryonic Kidney	1742	1	0.000574053
GSM2797120	AR	Breast	1744	1	0.000573394
ENCSR847LBF_2	FOXJ2	K562;Erythroblast;Bone Marrow	12212	7	0.000573207
GSM791408	BCAT1	LS180;Epithelium;Colon	5234	3	0.000573175
GSM1982474	HNF1A	Epithelium;Epididymis	13978	8	0.000572328
GSM1858623	ESR1	MCF-7;Epithelium;Breast	5242	3	0.000572301
GSM2086307	AR	VCaP;Epithelium;Prostate	5243	3	0.000572191
GSM2466478	ZNF136	293T	5246	3	0.000571864
GSM1517569	FLI1	A673;Polygonal;Muscle	3524	2	0.000567537
GSM634630	PPARG	SGBS;Adipocyte;Adipose	5289	3	0.000567215
GSM801052	CREB1	H3396;Epithelium;Mammary Gland	7064	4	0.000566251
GSM2311000	SPDEF	A549;Epithelium;Lung	3536	2	0.000565611
GSM1358400	AR	Prostate	23031	13	0.000564457
ENCSR449UFF_2	ZKSCAN1	MCF-7;Epithelium;Breast	1772	1	0.000564334
GSM2797124	AR	Breast	1774	1	0.000563698
GSM1643794	SOX2	Glioblastoma Stem Cell	1781	1	0.000561482
GSM1703567	PGR	Stromal Cell;Endometrium	14325	8	0.000558464
GSM2086298	AR	VCaP;Epithelium;Prostate	7163	4	0.000558425
GSM2432960	OTX2	D-341	39417	22	0.000558135
GSM1358408	AR	Prostate	17926	10	0.000557849
ENCSR086FZL_1	KAT8	K562;Erythroblast;Bone Marrow	1793	1	0.000557724
GSM1614034	TEAD4	MDA-MB-231;Epithelium;Breast	12560	7	0.000557325
GSM2026775	YY1	HEK293;Epithelium;Embryonic Kidney	5403	3	0.000555247
GSM935589	GATA2	SH-SY5Y;Bone Marrow	50625	28	0.000553086
GSM1358410	AR	Prostate	14465	8	0.000553059
GSM1447325	DMC1	Testis	14487	8	0.000552219

GSM1700641	ASCL1	NCI-H82	18171	10	0.000550327
GSM2419826	RELA	Detroit 562;Detroit 562 cells	7272	4	0.000550055
GSM2466477	ZNF135	293T	1824	1	0.000548246
GSM1260312	SOX11	GRANTA-519;B Lymphocyte	10949	6	0.000547995
GSM2817664	GATA2	Embryonic Stem Cell	9150	5	0.000546448
GSM2308950	ATF7IP	HeLa;Epithelium;Cervix	3668	2	0.000545256
GSM1551469	FOXA2	CyT49;Embryonic Stem Cell;Embryo	62432	34	0.000544593
GSM1505680	HNF1B	HUES64;Embryonic Stem Cell;Embryo	1838	1	0.00054407
GSM2086301	AR	VCaP;Epithelium;Prostate	11068	6	0.000542103

Supplementary Table 6: TFs of the STON1 gene region.

GSM_ID	Factor	Biosource	RP_score
GSM1358396	AR	Prostate	0.80923694
GSM817348	AR	VCaP;Epithelium;Prostate	0.717691
GSM2041108	RUNX2	Saos-2;Osteosarcoma cell	0.70346403
GSM1817200	TFAP2A	Neural crest cell;Cranial	0.6436058
GSM2537232	HOXB13	VCaP;Epithelium;Prostate	0.6428752
GSM1358400	AR	Prostate	0.64245087
GSM2401446	FOXA2	BJ;Fibroblast;Skin	0.64145106
GSM2537233	HOXB13	VCaP;Epithelium;Prostate	0.64141285
GSM2026843	ZNF384	HEK293;Epithelium;Embryonic Kidney	0.64019114
GSM2401466	FOXA2	BJ;Fibroblast;Skin	0.617796
GSM1354836	FOXA1	VCaP;Epithelium;Prostate	0.6024891
GSM1817197	TFAP2A	Neural crest cell;Cranial	0.5882079
GSM1358397	AR	Prostate	0.58524704
GSM1647188	MEF2B	HEK293A	0.5686849
GSM2977490	FOXA2	BJ;Fibroblast;Skin	0.5674158
GSM1358412	AR	Prostate	0.5630814
GSM817354	AR	VCaP;Epithelium;Prostate	0.5600972
GSM1700641	ASCL1	NCI-H82	0.5539697
GSM1071293	AR	Epithelium;Prostate	0.547317
GSM1777599	SUMO2	VCaP;Epithelium;Prostate	0.54712933
GSM2537231	HOXB13	VCaP;Epithelium;Prostate	0.5438745
GSM1817190	NR2F1	Neural crest cell;Cranial	0.5416917
GSM1358414	AR	Prostate	0.53995395
GSM1010738	GATA3	SK-N-SH;Neuroblastoma cell;Brain	0.53886425
GSM896987	KLF4	BJ;Fibroblast;Skin	0.53670436
GSM353651	AR	Epithelium;Prostate	0.5326601
GSM803497	RAD21	SK-N-SH_RA;Neuron;Brain	0.5318943
GSM1716764	HOXB13	LNCaP;Epithelium;Prostate	0.52144337
GSM803335	REST	SK-N-SH;Neuroblastoma cell;Brain	0.5190633
GSM1358413	AR	Prostate	0.5124689
GSM1122662	KDM5B	SUM185PE;Epithelium;Mammary Gland	0.50877196
GSM2537226	FOXA1	VCaP;Epithelium;Prostate	0.50389993
GSM1003631	MXI1	SK-N-SH;Neuroblastoma cell;Brain	0.49941087
GSM1358398	AR	Prostate	0.49383444
GSM1358411	AR	Prostate	0.49336636
GSM1647184	MEF2B	HEK293A	0.4912725
GSM817352	AR	VCaP;Epithelium;Prostate	0.48882034
GSM2977502	FOXA2	BJ;Fibroblast;Skin	0.48596323
GSM1518912	SMARCA4	501-Mel;Melanoma Cell	0.48118773
GSM1010901	JUND	SK-N-SH;Neuroblastoma cell;Brain	0.4808602
GSM999792	PR	Leiomyoma Cell	0.48019558
GSM2537227	FOXA1	VCaP;Epithelium;Prostate	0.47971213
GSM2543156	ZBTB48	U2OS	0.47814602
ENCSR206ETG_1	CTCF	gastroesophageal sphincter	0.47802603
GSM1358407	AR	Prostate	0.47632876
ENCSR431EHE_1	POLR2A	Sigmoid Colon	0.47324702
GSM1647185	MEF2B	HEK293A	0.46872485
GSM2537230	FOXA1	VCaP;Epithelium;Prostate	0.4687215
GSM869307	FOXO3	DL23;Epithelium;Colon	0.46866667
GSM651558	SMARCA4	CD36 Cell	0.4683191
GSM1777601	SUMO2	VCaP;Epithelium;Prostate	0.46252248
GSM1003522	EZH2	DND-41;T Lymphocyte;Blood	0.4624468
GSM1370274	IRF4	OCI-Ly3;B Lymphocyte;Bone Marrow	0.46011502
GSM2771534	ZBTB7A	K562;Erythroblast;Bone Marrow	0.45949364
GSM1010898	MAX	H1;Embryonic Stem Cell;Embryo	0.4580451
GSM733786	H2AZ	K562;Erythroblast;Bone Marrow	0.45696622
GSM2537229	FOXA1	VCaP;Epithelium;Prostate	0.45674282
GSM869313	POLR2A	DL23;Epithelium;Colon	0.45490453
GSM1336148	SNAI2	Keratinocyte	0.4516691

GSM1011562	TFAP2C	A375;Skin	0.4503965
GSM1010845	TEAD4	H1;Embryonic Stem Cell;Embryo	0.45014733
GSM1330728	POLR2A	MRC5;Fibroblast;Lung	0.44953552
GSM1856031	GATA3	MDA-MB-231;Epithelium;Mammary Gland	0.44876888
GSM1858643	FOXA1	ZR-75-1;Embryonic Stem Cell;Breast	0.4473559
GSM1817199	TFAP2A	Neural crest cell;Cranial	0.44624376
GSM1856029	GATA3	MDA-MB-231;Epithelium;Mammary Gland	0.44620165
ENCSR097EEA_1	POLR2A	gastroesophageal sphincter	0.44553298
GSM820430	TERC	HeLa;Epithelium;Cervix	0.4431006
GSM1358408	AR	Prostate	0.44286096
GSM2574752	SMAD4	HepG2;Epithelium;Liver	0.43911707
GSM1846896	KMT2D	Patient derived xenograft cells	0.43808633
GSM803523	MAX	K562;Erythroblast;Bone Marrow	0.43535253
GSM1716763	HOXB13	Prostate	0.4348979
GSM1598220	AR	Prostate	0.43409628
GSM817347	AR	VCaP;Epithelium;Prostate	0.4314898
GSM2537225	FOXA1	VCaP;Epithelium;Prostate	0.43136087
GSM2280027	TBX5	Cardiomyocyte	0.43047157
GSM2817667	TFAP2A	Embryonic Stem Cell	0.42892486
GSM820438	TERC	HeLa;Epithelium;Cervix	0.42840803
GSM820437	TERC	HeLa;Epithelium;Cervix	0.4276263
GSM2977504	FOXA2	BJ;Fibroblast;Skin	0.4272727
GSM1358404	AR	Prostate	0.42513168
GSM1495181	HSF1	U2OS;Bone	0.4245524
GSM1139038	SOX2	HCC95;Epithelium;Lung	0.4194432
GSM2771535	ZBTB7A	K562;Erythroblast;Bone Marrow	0.41499645
GSM1463464	FOXA1	VCaP;Epithelium;Prostate	0.4147412
GSM2401467	FOXA2	BJ;Fibroblast;Skin	0.41194335
GSM2466584	ZNF534	293T	0.4101689
GSM588927	TFAP2A	MCF-7;Epithelium;Breast	0.40889868
GSM2401447	FOXA2	BJ;Fibroblast;Skin	0.40842274
GSM2305319	RUNX2	MCF-7	0.4037482
GSM588928	TFAP2A	MCF-7;Epithelium;Breast	0.40184498
GSM1003546	H2AZ	A549;Epithelium;Lung	0.4007722
GSM1619040	KDM1A	NCI-H526;Epithelium;Lung	0.39960238
GSM889426	TFAP2C	BT-474;Epithelium;Mammary Gland	0.39928874
GSM1684632	POLR2A	Mesenchymal Stem Cell	0.39608496
GSM2029581	EP300	ZR-75-30	0.3960566
GSM2537234	HOXB13	VCaP;Epithelium;Prostate	0.39512992
GSM831039	RBBP5	H1;Embryonic Stem Cell;Embryo	0.39483082
GSM2432981	OTX2	D283 Med	0.3942838
GSM831035	KDM4A	H1;Embryonic Stem Cell;Embryo	0.39227107
GSM1003479	KDM4A	H1;Embryonic Stem Cell;Embryo	0.39227107
GSM2895354	H2AZ	K562;Erythroblast;Bone Marrow	0.39156625
GSM1888550	RUNX1	TF-1;Erythroblast;Bone Marrow	0.39151713
GSM749768	CTCF	WERI-Rb-1;Eye	0.3898252
GSM2537235	HOXB13	VCaP;Epithelium;Prostate	0.38860464
GSM733766	H2AZ	Osteoblast;Bone	0.3885993
GSM1071291	AR	Epithelium;Prostate	0.3870968
GSM1858640	FOXA1	ZR-75-1;Embryonic Stem Cell;Breast	0.38695827
GSM1922947	RBPJ	Neurosphere	0.38568085
GSM2242429	FOXA1	T47D;Breast	0.38556954
GSM1551468	FOXA2	CyT49;Embryonic Stem Cell;Embryo	0.38555413
GSM2394415	JUN	JHU-06;Endothelial Cell	0.38504755
ENCSR732DAQ_1	ZNF788	HEK293;Epithelium;Embryonic Kidney	0.3849105
GSM1622633	POLR2A	VCaP;Epithelium;Prostate	0.38449207
GSM935519	CEBPB	IMR90;Fibroblast;Lung	0.38411024
ENCSR777YSB_1	ZNF781	HEK293;Epithelium;Embryonic Kidney	0.38222224
ENCSR217QCK_2	ZNF697	HEK293;Epithelium;Embryonic Kidney	0.38222224
GSM1858641	FOXA1	ZR-75-1;Embryonic Stem Cell;Breast	0.38134205

GSM1777606	SUMO2	VCaP;Epithelium;Prostate	0.38117212
GSM1010900	TCF12	SK-N-SH;Neuroblastoma cell;Brain	0.3805716
GSM1354837	FOXA1	VCaP;Epithelium;Prostate	0.38052964
GSM1701828	SOX2	Neuronal Progenitor cell	0.38048682
ENCSR587OQL_1	SMARCA4	K562;Erythroblast;Bone Marrow	0.3800308
GSM1291199	TAL1	Cord blood	0.3798567
GSM798425	ESR1	MCF-7;Epithelium;Breast	0.37932184
ENCSR464KFG_2	ZNF140	HEK293;Epithelium;Embryonic Kidney	0.3767544
GSM2436682	MKL2	A673;Polygonal;Muscle	0.3757116
GSM1858644	FOXA1	ZR-75-1;Embryonic Stem Cell;Breast	0.37512437
GSM1024799	AHR	MCF-7;Epithelium;Breast	0.37444404
GSM2612451	ERF	VCaP;Epithelium;Prostate	0.37411663
GSM2817659	GATA3	H9;Embryonic Stem Cell;Embryo	0.3739965
GSM986067	GATA3	MCF-7;Epithelium;Breast	0.37379542
GSM1037516	POLR2A	DL23;Epithelium;Colon	0.37337133
GSM2664369	PHOX2B	CLB-Ga;Neuroblastoma cell	0.37309912
GSM2026854	ZNF317	HEK293;Epithelium;Embryonic Kidney	0.3729207
GSM763402	BCL6	OCI-Ly1;B cell lymphoma;Bone Marrow	0.37290925
GSM1622636	POLR2A	VCaP;Epithelium;Prostate	0.37231782
GSM803409	FOXA1	T47D;Epithelium;Mammary Gland	0.37219945
GSM1099031	FOXA1	MDA-MB-453;Epithelium;Mammary Gland	0.370973
GSM1071287	AR	Epithelium;Prostate	0.36510336
GSM2026874	ZBTB26	HEK293;Epithelium;Embryonic Kidney	0.3648431
GSM1858636	ESR1	ZR-75-1;Embryonic Stem Cell;Breast	0.36483008
GSM1187229	ASCL1	Fibroblast	0.36353946
GSM1068137	FOXA1	LNCaP;Epithelium;Prostate	0.36249614
GSM945604	IRF5	Monocyte;Blood	0.36137646
GSM2519950	KLF15	Muscle	0.36126885
GSM1556928	ESR1	LY2;Breast	0.36060235
GSM2430681	FOXA2	BJ;Fibroblast;Skin	0.3602638
GSM1858635	ESR1	ZR-75-1;Breast Cancer Cell;Breast	0.3596848
GSM1003500	H2AZ	Epithelium;Mammary Gland	0.35885167
ENCSR057MWG_2	H2AFZ	MCF-7;Epithelium;Breast	0.35724506
GSM2809636	H2AZ	HCT-116;Colon	0.3572062
ENCSR462FWS_2	ZNF101	HEK293;Epithelium;Embryonic Kidney	0.35705367
GSM1703567	PGR	Stromal Cell;Endometrium	0.35702693
GSM1139035	SOX2	TT;Thyroid	0.35650483
GSM1684633	POLR2A	Mesenchymal Stem Cell	0.35565087
GSM2359438	BRD4	A375;melanoma;Skin	0.3554448
GSM1019125	ESR1	MCF-7;Epithelium;Breast	0.3535503
GSM798423	ESR1	MCF-7;Epithelium;Breast	0.3535503
ENCSR777YSB_2	ZNF781	HEK293;Epithelium;Embryonic Kidney	0.35274816
GSM1003580	H2AZ	A549;Epithelium;Lung	0.352558
GSM817353	AR	VCaP;Epithelium;Prostate	0.35251957
GSM894102	MYC	H2171;Epithelium;Lung	0.35196725
ENCSR948QLZ_2	CBX1	K562;Erythroblast;Bone Marrow	0.35185188
GSM1010887	PBX3	SK-N-SH;Neuroblastoma cell;Brain	0.3504311
GSM1622626	SUMO2	VCaP;Epithelium;Prostate	0.34957722
GSM2769057	PAF1	DLD-1	0.3486379
GSM803495	EP300	SK-N-SH_RA;Neuron;Brain	0.3484646
GSM2029587	KDM5C	ZR-75-30	0.3482681
GSM1291197	TAL1	Cord blood	0.34783226
ENCSR477OJI_2	ZNF423	HEK293;Epithelium;Embryonic Kidney	0.34761235
GSM869314	POLR2A	DL23;Epithelium;Colon	0.34725386
GSM1354831	AR	VCaP;Epithelium;Prostate	0.34722778
GSM798439	FOXA1	MCF-7;Epithelium;Breast	0.34575263
GSM1327160	EMX1	293FT;Embryonic Kidney	0.34552994
GSM2546254	SMARCE1	HMLE-Twist-ER_125nM	0.34392458
GSM1898103	SATB1	SH-SY5Y;Neuron	0.34385663
GSM1462477	EGLN2	T47D;Epithelium;Mammary Gland	0.34375

GSM2740928	SMARCA4	HepG2;Epithelium;Liver	0.34332988
GSM2817666	TFAP2A	Embryonic Stem Cell	0.34325686
GSM1858655	FOXA1	T47D;Embryonic Stem Cell;Breast	0.34245753
GSM1858642	FOXA1	ZR-75-1;Embryonic Stem Cell;Breast	0.34217748
GSM1858625	FOXA1	MCF-7;Epithelium;Breast	0.3420396
GSM2065882	MYC	HCT-116;Colon cancer cell;Colon	0.34181902
ENCSR369NGL_1	POLR2AphosphoS5	iPSC	0.34174392
GSM1703607	FOXO1	Stromal Cell;Endometrium	0.34142458
GSM1257396	NR2F2	Stromal Cell	0.34104523
GSM1856030	GATA3	MDA-MB-231;Epithelium;Mammary Gland	0.34097767
GSM2026856	ZNF382	HEK293;Epithelium;Embryonic Kidney	0.34091744
GSM2771541	ZBTB7A	HUDEP-2	0.34042552
GSM1463466	POLR2A	VCaP;Epithelium;Prostate	0.3399894
GSM1003633	CTCF	SK-N-SH;Neuroblastoma cell;Brain	0.33967632
GSM1858639	FOXA1	ZR-75-1;Embryonic Stem Cell;Breast	0.33933443
GSM2257819	ESR1	MCF-7;Breast	0.33931518
GSM1122848	FAIRE	Epithelium;Mammary Gland	0.33906853
GSM2068363	DUX4	LHCN-M2;Myoblast;Skeletal Muscle	0.3389953
GSM1336157	SNAI2	Keratinocyte	0.33789027
GSM1405131	ERCC6	Fibroblast;Skin	0.33787003
GSM1777604	SUMO2	VCaP;Epithelium;Prostate	0.33763874

Supplementary Table 7: TFs of the FSHR gene region.

GSM_ID	Factor	Biosource	RP_score
GSM1505663	GATA6	HUES64;Embryonic Stem Cell;Embryo	0.67335004
GSM837613	DUX4	Myoblast;Muscle	0.58326757
GSM542547	ESR1	U2OS;Bone	0.571045
GSM791413	CDX2	LS180;Epithelium;Colon	0.5519435
GSM1567046	OTX2	6540 cells;Epithelium	0.50924367
GSM1684643	CEBPB	Mesenchymal Stem Cell	0.47663707
GSM727589	FOXH1	H9;Embryonic Stem Cell;Embryo	0.46360317
GSM2635693	TEAD4	H1;Embryonic Stem Cell;Embryo	0.4608223
GSM791414	CDX2	LS180;Epithelium;Colon	0.4412586
GSM1010803	POLR2A	H1;Embryonic Stem Cell;Embryo	0.43115166
GSM1517751	MITF	501-Mel;Melanoma Cell	0.42418155
GSM2664369	PHOX2B	CLB-Ga;Neuroblastoma cell	0.41740957
GSM2466504	ZNF248	293T	0.41265947
GSM1505632	FOXA1	HUES64;Embryonic Stem Cell;Embryo	0.39434308
GSM1684635	CEBPB	Mesenchymal Stem Cell	0.39138296
GSM1071287	AR	Epithelium;Prostate	0.36510336
GSM640691	EOMES	H9;Embryonic Stem Cell;Embryo	0.3519425
GSM1542533	YAP1	NCI-H2052;Epithelium;Lung	0.34773132
GSM2232927	TEAD4	BE2-C;Neuroblastoma cell;Bone Marrow	0.34234023
GSM1684642	CEBPB	Mesenchymal Stem Cell	0.34169152
GSM542548	ESR1	U2OS;Bone	0.33721936
GSM1302192	CDK2	T47D-MTVL;Epithelium;Mammary Gland	0.32709196
GSM1682262	AGO2	K562;Erythroblast;Bone Marrow	0.31393862
GSM935306	MAFF	HepG2;Epithelium;Liver	0.30560336
GSM2665569	NKX2-1	hESC	0.30526316
GSM727584	SMAD2/3	H9;Embryonic Stem Cell;Embryo	0.3003942
GSM2050700	GATA6	Endoderm Cell;Endoderm	0.29590946
GSM1483191	NELFE	H9;Embryonic Stem Cell;Embryo	0.2956585
GSM1505742	SMAD1	HUES64;Embryonic Stem Cell;Embryo	0.2933738
GSM1505741	SMAD1	HUES64;Embryonic Stem Cell;Embryo	0.28655836
GSM945604	IRF5	Monocyte;Blood	0.28430307
GSM1551469	FOXA2	CyT49;Embryonic Stem Cell;Embryo	0.2789148
GSM791412	CEBPB	LS180;Epithelium;Colon	0.27326825
GSM935305	MAFK	HepG2;Epithelium;Liver	0.27282932
GSM1316337	PDX1	CyT49;Embryonic Stem Cell;Embryo	0.2690741
GSM651557	SMARCA4	CD36 Cell	0.26513138
GSM1266817	SMAD2	WA09;Embryonic Stem Cell;Embryo	0.261753
GSM1684641	CEBPB	Mesenchymal Stem Cell	0.25975546
GSM1483190	NELFE	H9;Embryonic Stem Cell;Embryo	0.25515234
GSM2257824	FOXA1	MCF-7;Breast	0.24525833
GSM727564	FOXH1	H9;Embryonic Stem Cell;Embryo	0.24384674
GSM1684647	CEBPB	Mesenchymal Stem Cell	0.24100946
GSM803529	JUND	H1;Embryonic Stem Cell;Embryo	0.24089406
GSM2410317	EBF3	SK-N-SH;Neuroblastoma cell;Brain	0.23882452
GSM1684636	CEBPB	Mesenchymal Stem Cell	0.23632993
GSM2409664	SMARCB1	G-401;Malignant rhabdoid tumor	0.23121743
GSM1370293	SPIB	OCI-Ly3;B Lymphocyte;Bone Marrow	0.2309524
GSM803466	RAD21	H1;Embryonic Stem Cell;Embryo	0.23013194
GSM1197322	GATA6	LS174T;Epithelium;Colon	0.22735101
GSM641426	ESR1	U2OS;Bone	0.22460894
GSM602291	EP300	H9;Embryonic Stem Cell;Embryo	0.22265624
GSM1113434	REPIN1	Kasumi-1;Myeloblast;Blood	0.2215591
GSM1416931	PDX1	Embryonic Stem Cell;Embryo	0.21831147
GSM1197321	GATA6	LS174T;Epithelium;Colon	0.21807268
GSM791404	VDR	LS180;Epithelium;Colon	0.21787113
GSM1505706	OTX2	HUES64;Embryonic Stem Cell;Embryo	0.21294719
GSM1082931	POLR2A	T Lymphocyte;Blood	0.2124752
GSM1370281	SPIB	OCI-Ly10;B Lymphocyte;Bone Marrow	0.21195549
GSM1602666	GATA3	KELLY	0.20982143

GSM1250900	GATA4	KATO III;Stomach	0.2096837
GSM1003626	EP300	SK-N-SH;Neuroblastoma cell;Brain	0.20955671
GSM1010845	TEAD4	H1;Embryonic Stem Cell;Embryo	0.2071639
GSM935380	USF2	H1;Embryonic Stem Cell;Embryo	0.2067039
GSM2550230	EP300	Stem cell	0.20518412
GSM1693100	MAML3	SK-N-SH;Neuroblastoma cell;Brain	0.2038345
GSM1197320	GATA6	LS174T;Epithelium;Colon	0.20024161
GSM2257817	CTCF	MCF-7;Breast	0.19718868
GSM791411	CEBPB	LS180;Epithelium;Colon	0.19530883
GSM1336148	SNAI2	Keratinocyte	0.19506532
GSM2409665	SMARCC1	G-401;Malignant rhabdoid tumor	0.19463779
GSM820430	TERC	HeLa;Epithelium;Cervix	0.19384277
GSM1370289	SPIB	OCI-Ly3;B Lymphocyte;Bone Marrow	0.18782027
GSM2026853	ZIM3	HEK293;Epithelium;Embryonic Kidney	0.18594721
GSM820434	HOTAIR	MDA-231;Epithelium;Mammary Gland	0.18537512
GSM935295	CEBPB	H1;Embryonic Stem Cell;Embryo	0.18534483
GSM1370274	IRF4	OCI-Ly3;B Lymphocyte;Bone Marrow	0.1845131
GSM651558	SMARCA4	CD36 Cell	0.17908733
GSM2257818	CTCF	MCF-7;Breast	0.1786325
GSM2543157	ZBTB48	U2OS	0.17769295
GSM518374	NANOG	H1;Embryonic Stem Cell;Embryo	0.1736627
GSM1970164	SOX4	Fibroblast;Fetal Lung	0.17081259
GSM803426	USF1	H1;Embryonic Stem Cell;Embryo	0.17010045
GSM820437	TERC	HeLa;Epithelium;Cervix	0.16835861
GSM1198993	SOX2	ReN-VM;Neural Stem Cell	0.16826062
GSM743039	CDX2	COLO-320;Epithelium;Colon	0.16577381
GSM2629527	MYOG	H9;Embryonic Stem Cell;Embryo	0.16099477
GSM1680102	GATA3	BE2-C;Neuroblast;Brain	0.1577374
GSM798394	ESR1	Epithelium;Mammary Gland	0.1568228
GSM1816090	SPI1	Hematopoietic stem and progenitor cell;Bone Marrow	0.15480226
GSM1542532	YAP1	NCI-H2052;Epithelium;Lung	0.15457474
GSM2449944	PITX3	SH-SY5Y;Neuron;Midbrain	0.15406261
GSM959051	ZNF143	FLP143HA;Epithelium;Embryonic Kidney	0.15313098
GSM1505704	OTX2	HUES64;Embryonic Stem Cell;Embryo	0.15292916
GSM1024799	AHR	MCF-7;Epithelium;Breast	0.15292507
GSM935610	MAFK	HepG2;Epithelium;Liver	0.1521573
GSM1684645	CEBPB	Mesenchymal Stem Cell	0.1518962
GSM2466457	ZIM3	293T	0.15135306
ENCSR445ACU_1	SOX13	HepG2;Epithelium;Liver	0.15090424
GSM822289	CTCF	A549;Epithelium;Lung	0.14949425
GSM2550216	EP300	Fibroblast	0.14458725
GSM2432984	NEUROD1	D283 Med	0.14400174
GSM803495	EP300	SK-N-SH_RA;Neuron;Brain	0.14285713
GSM2257820	ESR1	MCF-7;Breast	0.1409492
GSM2574801	NFATC3	HepG2;Epithelium;Liver	0.14049587
GSM2432944	NEUROD1	D283 Med	0.13915046
GSM2543160	ZBTB48	U2OS	0.13906072
GSM2432971	OTX2	D-341	0.13883972
GSM727561	SMAD4	H9;Embryonic Stem Cell;Embryo	0.13798183
GSM1370275	SPI1	OCI-Ly3;B Lymphocyte;Bone Marrow	0.13760526
GSM945611	STAT4	Monocyte;Blood	0.13501751
GSM1421017	SPI1	Monocyte	0.13219675
GSM2664370	GATA3	CLB-Ga;Neuroblastoma cell	0.13071105
GSM970828	CTCF	BCBL1;B cell lymphoma	0.13046646
GSM935567	GATA3	SH-SY5Y;Bone Marrow	0.13026355
GSM2432951	OTX2	D-341	0.12925898
GSM1841308	SPI1	RS4;B Lymphocyte	0.12919557
GSM1250896	GATA4	AGS;Epithelium;Stomach	0.12684219
GSM822288	POLR2A	A549;Epithelium;Lung	0.12606697
GSM2432982	NEUROD1	D283 Med	0.12508897

GSM1081542	SMC3	HEK293T;Epithelium;Embryonic Kidney	0.124524996
GSM2432950	OTX2	D-341	0.12443438
GSM2432942	OTX2	D283 Med	0.12358992
GSM2432967	OTX2	D-341	0.1224267
GSM2432981	OTX2	D283 Med	0.12184523
GSM1082927	H2AZ	T Lymphocyte;Blood	0.12156558
GSM2432980	OTX2	D283 Med	0.12036324
GSM2432983	NEUROD1	D283 Med	0.1200453
GSM1334007	SPI1	GM12878;B Lymphocyte;Blood	0.11973993
GSM594603	ESR1	MCF-7;Epithelium;Breast	0.11926033
GSM2432943	OTX2	D283 Med	0.11917264
GSM1010837	NR2F2	MCF-7;Epithelium;Breast	0.11896279
GSM2550259	POLR2A	Fibroblast	0.11842651
GSM1354831	AR	VCaP;Epithelium;Prostate	0.11547667
GSM1336147	SNAI2	Keratinocyte	0.11476436
GSM1354836	FOXA1	VCaP;Epithelium;Prostate	0.11470985
GSM1358819	HIRA	IMR90;Fibroblast;Lung	0.11423142
GSM733760	H2AZ	Myoblast;Skeletal Muscle	0.11371073
GSM1544114	MBD2	HMLER	0.113659024
GSM2474865	MCM2	HCT-116;HCT116	0.11319703
GSM651543	CTCF	Proerythroblast;Bone Marrow	0.11214954
GSM1234010	CTCF	GM18526;Lymphoblastoid;Blood	0.11211854
GSM2432962	OTX2	D-341	0.111181304
GSM980658	AR	VCaP;Epithelium;Prostate	0.11079828
GSM1010783	GATA3	MCF-7;Epithelium;Breast	0.110740736
GSM980657	AR	VCaP;Epithelium;Prostate	0.110687025
GSM2550258	EP300	Fibroblast	0.110396974
GSM1370285	SPIB	H929;B Lymphocyte;Bone Marrow	0.11008798
GSM1370284	SPI1	H929;B Lymphocyte;Bone Marrow	0.10990877
GSM2467294	ESR1	endometrioid adenocarcinoma	0.109698325
GSM1816089	SPI1	Hematopoietic stem and progenitor cell;Fetal Liver	0.10826709
GSM1917730	EP300	PC-3;Epithelium;Prostate	0.10688836
GSM803353	FOXP2	SK-N-MC;Brain	0.106181346
GSM1463464	FOXA1	VCaP;Epithelium;Prostate	0.10610559
GSM803365	REST	H1;Embryonic Stem Cell;Embryo	0.105274685
GSM803531	SPI1	GM12878;B Lymphocyte;Blood	0.10515119
GSM726984	ERG	K562;Erythroblast;Bone Marrow	0.104034625
GSM1542271	KDM1A	SH-SY5Y;Bone Marrow	0.10380539
GSM803538	BATF	GM12878;B Lymphocyte;Blood	0.10340502
GSM1551466	FOXA1	CyT49;Embryonic Stem Cell;Embryo	0.10319666
GSM822304	MYC	MCF-7;Epithelium;Breast	0.102413066
GSM696842	AR	VCaP;Epithelium;Prostate	0.101793244
GSM986067	GATA3	MCF-7;Epithelium;Breast	0.101606086
GSM803398	SPI1	GM12891;Lymphoblastoid;Blood	0.10088596
GSM1782712	SPI1	K562;Erythroblast;Bone Marrow	0.10020917
GSM935589	GATA2	SH-SY5Y;Bone Marrow	0.100101516
GSM798425	ESR1	MCF-7;Epithelium;Breast	0.10006649
GSM1716768	HOXB13	LHSAR;Epithelium;Prostate	0.09935533
GSM1556334	RELB	L1236;Hodgkin Lymphoma Cell	0.098838985
GSM1684646	CEBPB	Mesenchymal Stem Cell	0.09789194
GSM1045472	EED	DU145;Epithelium;Prostate	0.09766494
GSM1370276	SPIB	OCI-Ly3;B Lymphocyte;Bone Marrow	0.09736308
GSM798437	FOXA1	MCF-7;Epithelium;Breast	0.09704641
GSM1577746	STAT5B	T Lymphocyte;Blood	0.09646922
GSM1006865	POLR2A	MCF-7;Epithelium;Breast	0.09641125
GSM1505637	FOXA2	HUES64;Embryonic Stem Cell;Embryo	0.09617401
GSM1010738	GATA3	SK-N-SH;Neuroblastoma cell;Brain	0.09581218
GSM1505662	GATA6	HUES64;Embryonic Stem Cell;Embryo	0.09435708
GSM2235689	AR	VCaP;Epithelium;Prostate	0.09402262
GSM1234025	STAG1	GM18526;Lymphoblastoid;Blood	0.09380282

GSM1607526	NR3C1	U2OS;Bone	0.093525186
GSM1551465	FOXA1	CyT49;Embryonic Stem Cell;Embryo	0.09348613
GSM1463460	AR	VCaP;Epithelium;Prostate	0.09258385
GSM1527034	AR	CWR22Pc;Epithelium;Prostate	0.09235818
GSM1505696	NANOG	HUES64;Embryonic Stem Cell;Embryo	0.09069549
GSM1080932	HDAC1	Nalm6;B cell precursor;Blood	0.09055779
ENCSR264RJX_2	POU5F1	iPSC	0.089907445
GSM607951	RUNX1	Megakaryocyte;Cord blood	0.08969823
GSM1556336	NFKB1	L1236;Hodgkin Lymphoma Cell	0.088086985
GSM1122651	KDM5B	MCF-7;Epithelium;Breast	0.088031046
GSM2364545	AFF1	SEM;B Lymphocyte	0.08792671
GSM545203	POU5F1	H9;Embryonic Stem Cell;Embryo	0.087893866
GSM803344	REST	HepG2;Epithelium;Liver	0.087789916
GSM803384	SPI1	K562;Erythroblast;Bone Marrow	0.08675342
GSM1701825	SOX2	hESC	0.08668243
GSM1681425	SPI1	Macrophage;Blood	0.08587196
GSM2279971	TBX5	Cardiomyocyte	0.08472086
GSM822277	CTCF	GM19239;Lymphoblastoid;Blood	0.08259823
GSM1234026	STAG1	GM18526;Lymphoblastoid;Blood	0.08226285
GSM1577749	STAT5B	T Lymphocyte;Blood	0.082188696
GSM2332315	LARP7	HeLa;Epithelium;Cervix	0.08124547
